# A hydrogen-bonded conjugated quinone polymer enables ultrafast and ultrastable NH_4_^+^ storage for aqueous ammonium-ion batteries

**DOI:** 10.1039/d6sc04614b

**Published:** 2026-07-17

**Authors:** Xingru Chen, Rong Ge, Xueqing Ren, Wei Qin, Yu Ge, Junyue Luo, Guangzheng Xu, Jiamin Zhang, Beibei Yang, Yongzheng Zhang, Duan Bin, Hongbin Lu, Yonggang Wang

**Affiliations:** a Department of Polymer Materials and Science, College of Chemistry and Chemical Engineering, Nantong University Nantong 226019 China dbin17@fudan.edu.cn; b Haian Institute of High-end Textile, Nantong University Nantong 226000 P R China luhb@nju.edu.cn; c Department of Chemistry and Shanghai Key Laboratory of Molecular Catalysis and Innovative Materials, Institute of New Energy, iChEM (Collaborative Innovation Center of Chemistry for Energy Materials), Fudan University Shanghai 200433 China ygwang@fudan.edu.cn

## Abstract

Quinone-based organic compounds are attractive electrodes for aqueous ammonium-ion batteries (AAIBs), whereas the strong hydrogen-bond interactions between C=O active sites and water molecules readily cause active-material dissolution, and they also intrinsically suffer from sluggish reaction kinetics. Herein, an n-type organic polymer (HMND) is designed by employing benzoquinone units as redox-active centers for NH_4_^+^ storage and simultaneously constructing an intramolecular hydrogen-bonding network to overcome these limitations. This architecture enhances structural stability and extends π-conjugation, enabling efficient electron delocalization and improved accessibility of active sites. Crucially, the hydrogen-bonding network facilitates a unique NH_4_^+^ transport pathway, where NH_4_^+^ ions migrate along the molecular edges with low energy barriers *via* hydrogen-bond interactions. Consequently, the HMND anode delivers high capacity and excellent rate capability (200.7 mAh g^−1^ at 0.5 A g^−1^ and 116.3 mAh g^−1^ at 120 A g^−1^), along with remarkable cycling stability (82.81% retention after 10 000 cycles at 5 A g^−1^). The HMND//MnO@C full battery also exhibits durable performance, maintaining 108 mAh g^−1^ over 10 000 cycles. Experimental and theoretical analyses reveal a two-step NH_4_^+^ storage mechanism, where NH_4_^+^ ions sequentially attack the two distinct C=O sites along the molecular edge, achieving reversible electron storage through the formation and cleavage of N–H⋯O hydrogen bonds.

## Abstract 2

A hydrogen-bonded conjugated polymer (HMND) is designed as the anode to overcome dissolution and enable ultrafast NH_4_^+^ storage in aqueous ammonium-ion batteries.

## Graphical abstract

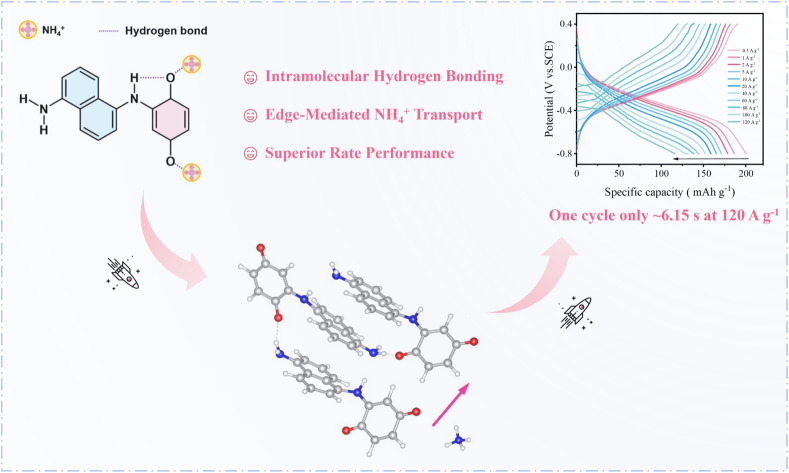

## Introduction

Aqueous batteries have garnered significant attention due to the inherent non-flammability, high ionic conductivity, and environmental friendliness of their electrolytes. Among these, aqueous ammonium-ion batteries (AAIBs) have emerged as a particularly promising system.^1–4^ Compared to metal ions (*e.g.*, Li^+^, Na^+^, Zn^2+^, *etc*.), ammonium ions (NH_4_^+^) offer distinct advantages, including a small hydrated radius, low molar mass, and rapid diffusion kinetics.^5^ The tetrahedral geometry centered on a nitrogen atom enables NH_4_^+^ to interact with host materials *via* hydrogen bonding, which enhances reaction reversibility and structural stability.^6,7^ For these advantages, considerable efforts have been devoted to exploring suitable electrode materials for AAIBs, with the aim of designing advanced and sustainable aqueous battery chemistries.

To date, inorganic anode materials have been employed for reversible NH_4_^+^ storage, including amorphous oxides and transition metal oxides/sulfides.^8–10^ However, these materials often suffer from low specific capacity, significant volume expansion, and insufficient cycling stability.^11^ Beyond inorganic materials, organic electrodes with C=O or C=N bonds have emerged as promising candidates due to their structural tunability, abundant redox-active sites, and favorable environmental compatibility.^12,13^ Unfortunately, their practical applications have been severely hindered by issues such as dissolution in aqueous electrolyte and low conductivity, which cause the poor cycling performance and sluggish kinetics. To tackle these issues, extensive efforts have been devoted to strategies such as polymerizing soluble small molecules into anti-dissolution polymers or conjugated structures for developing stable AAIBs.^14–16^ For instance, Bao *et al.* designed a carbonyl-based covalent organic framework that achieves good cycling stability up to 8000 cycles at 6 A g^−1^ while retaining high specific capacity of 141 mAh g^−1^ at 0.1 A g^−1^.^17^ Yan *et al.* developed an organic poly(1,5-naphthalenediamine) anode that retained a capacity of ∼47 mAh g^−1^ at a super-high current density of 50 A g^−1^ and demonstrated good cycling stability with 94% capacity retention after 1000 cycles at 5 A g^−1^.^18^ These advancements broaden the design of organic molecules to improve the stability, specific capacity or rate performance, while they are difficult to complement each other to realize both targets simultaneously. Specifically, most of the reported organic electrodes for AAIBs could deliver a high specific discharge capacity or high stability up to 8000 cycles, whereas most still suffer from unsatisfactory large-current tolerance (<50 A g^−1^) caused by slow electron delocalization. This has driven further efforts to develop organic electrodes for rapid NH_4_^+^ storage while simultaneously avoiding the instability caused by dissolution in aqueous electrolyte. Recent advances have introduced intramolecular hydrogen bonding (IHB) to equilibrate charge density at active sites and prevent intermolecular hydrogen bonding with water molecules, thereby redistributing charges along molecular backbones and rendering organic small molecules poorly soluble.^19,20^ Meanwhile, quinone-based organic compounds featuring IHB can enhance charge delocalization to boost redox kinetics during the charge–discharge process. Therefore, constructing IHB in C=O polymers is regarded as an effective strategy to simultaneously achieve high-rate capability and long-term cycling stability.

Building on these considerations, an n-type organic polymer (HMND), designed by integrating benzoquinone units (C=O bonds) with a 1,5-diaminonaphthalene linker (electron-donating –NH_2_ group), is developed as the anode material for AAIBs. Benefiting from the distinct IHB and extended π-conjugated backbone, the HMND structure is effectively stabilized, enhancing charge delocalization and electronic transport. When employed as an anode, the HMND exhibits exceptional rate capability and cycling stability for NH_4_^+^ storage. Specifically, the HMND electrode delivers a specific capacity of 116.3 mAh g^−1^ even at an ultrahigh current density of 120 A g^−1^ and retains 82.81% of its capacity after 10 000 cycles at 5 A g^−1^. Theoretical simulations reveal that NH_4_^+^ transport preferentially occurs along the molecular edges and perpendicular to the layered planes rather than through the interlayer spaces, which is more favorable for maintaining fast redox kinetics and structural stability. *Ex situ* FTIR and Raman verify the reversible C=O/C–O conversion, while DFT calculations confirm a two-step, two-electron NH_4_^+^ storage mechanism. When coupled with a MnO@C cathode, the assembled HMND//MnO@C full cell achieves a high specific capacity of 151.6 mAh g^−1^ at 1 A g^−1^, with a remarkable capacity retention of 81.6% over 10 000 cycles at 5 A g^−1^. This work presents an effective material design strategy that simultaneously addresses the dissolution issue of organic electrodes and optimizes ion transport pathways through molecular engineering, paving the way for high-capacity, long-life AAIBs.

## Results and discussion

**Fig. 1:**
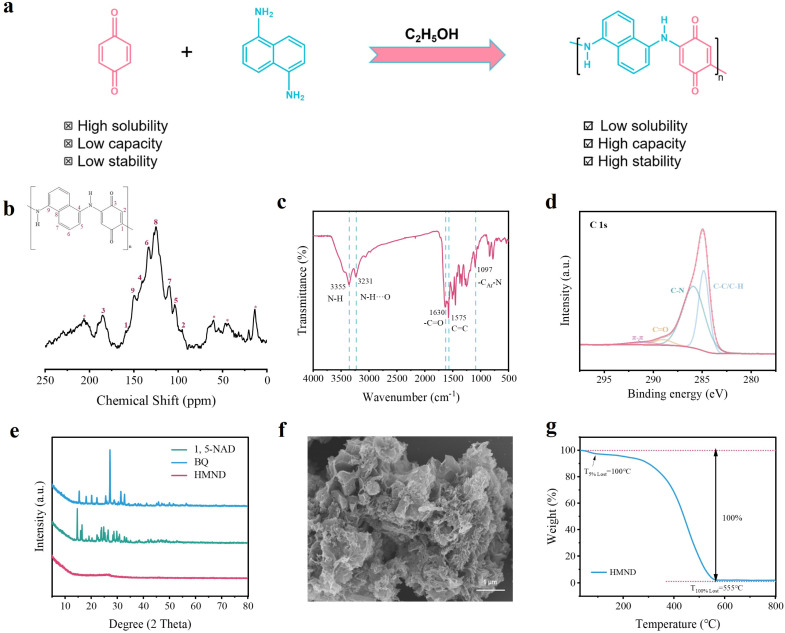
Synthesis and characterization studies. (a) Synthetic route and the molecular structure of HMND. (b) Solid-state ^13^C NMR spectrum of HMND. (c) FTIR spectrum of HMND. (d) High-resolution C 1s XPS spectra of HMND. (e) XRD patterns of HMND and its precursor materials. (f) SEM image of HMND. (g) Thermogravimetric analysis (TGA) curve of HMND.

The HMND was synthesized by a condensation reaction using 1,5-diaminonaphthalene (1,5-NAD) and *p*-benzoquinone (BQ) as starting materials in ethanol solvent, which facilitates homogeneous mixing of the precursors while also weakening electrostatic repulsion between the reactive groups to promote the formation of intramolecular C=O⋯N–H hydrogen bonds, as illustrated in Fig. 1a. The molecular structure of HMND was confirmed by solid-state ^13^C NMR spectroscopy (Fig. 1b). The spectrum shows that the signals in the range of 100–150 ppm are assigned to the C–C bonds (carbon atoms 4–8) of the naphthalene ring, while the characteristic peak at 170 ppm is attributed to the carbonyl carbon (carbon atom 3) of the C=O group.^21^ Fourier-transform infrared (FT-IR) spectroscopy provided further support for the molecular structure analysis. As shown in Fig. S1 and 1c, the characteristic N–H stretching bands of the precursor 1,5-NAD remained at 3355 cm^−1^ and 3231 cm^−1^ in HMND, while the C=O stretching vibration shifted from 1656 cm^−1^ in BQ to 1630 cm^−1^ in HMND, indicating an altered chemical environment of the carbonyl group during the reaction. Meanwhile, the characteristic band at 1097 cm^−1^, corresponding to the –C_Ar_–N stretching vibration, persisted throughout the reaction. The high-resolution C 1s XPS spectrum of HMND powder (Fig. 1d) exhibited four characteristic peaks at binding energies of 284.80 eV, 285.98 eV, 289.08 eV and 291.18 eV, which are assigned to C–C/C–H, C–N, and C=O functional groups, and a π–π satellite peak, respectively.^22^ Furthermore, the N 1s and O 1s spectra (Fig. S2) confirmed the presence of N–H and C=O bonds in HMND. These spectral changes collectively confirm the successful synthesis of HMND. The X-ray diffraction (XRD) pattern of HMND (Fig. 1e) shows no distinct diffraction peaks across the entire scanning range, but instead exhibits a broad peak centered at ∼28°, indicating its amorphous nature. This behavior originates from polymerization-induced disruption of intermolecular packing, which suppresses long-range order and results in structural disorder.^23^ Such a disordered structure typically exposes more surface and edge sites, thereby facilitating thorough electrolyte infiltration and rapid ion accessibility.^24^ HRTEM imaging (Fig. S3) further confirmed the amorphous nature of HMND, consistent with the XRD result. The scanning electron microscopy (SEM) image (Fig. 1f) reveals that the synthesized HMND exhibits a structure composed of numerous thin and curved sheet-like units. Energy-dispersive X-ray spectroscopy (EDS) elemental mapping (Fig. S4) demonstrated the uniform spatial distribution of C, N, and O throughout the entire material.

According to the thermogravimetric analysis (TGA) curves (Fig. 1g), the HMND showed almost no mass loss below 100 °C, indicating superior structural rigidity. The first significant mass loss began at 100 °C (corresponding to 5% mass loss), which is primarily attributed to the removal of surface-adsorbed water rather than bulk material decomposition, confirming its intrinsic safety within conventional battery operating temperature ranges. When the temperature increased to 555 °C, nearly 100% mass loss was observed, suggesting complete decomposition or carbonization. Its thermal tolerance significantly exceeds that of most organic electrode materials,^25^ which may be related to its polymerized structure constructed from naphthoquinone and aromatic units. The inherent correlation between thermal stability and electrochemical durability establishes a clear design paradigm for developing highly stable, long-life organic electrode materials.

To identify the optimal electrolyte system for ammonium-ion storage in HMND, its electrochemical performance was systematically evaluated in 1 M NH_4_Ac, 0.5 M (NH_4_)_2_SO_4_, and 1 M NH_4_Cl solution, as shown in Fig. S5. Cyclic voltammetry (CV) tests revealed that HMND in the NH_4_Ac electrolyte exhibited the largest integrated CV curve area, indicating the highest reversible capacity (Fig. S5d–f). Rate capability tests (Fig. S5h) further confirmed this trend; HMND delivered a specific capacity of 192.5 mAh g^−1^ at 0.5 A g^−1^ in NH_4_Ac, significantly outperforming that in (NH_4_)_2_SO_4_ and NH_4_Cl. Moreover, HMND demonstrated good cycling stability in both NH_4_Ac and (NH_4_)_2_SO_4_ electrolytes (Fig. S5g), but underwent more rapid degradation in NH_4_Cl, retaining only 66.73% of its capacity after 10 000 cycles. The ionic conductivities of the three electrolytes were measured by electrochemical impedance spectroscopy (EIS), yielding values of 160.92 mS cm^−1^ for 1 M NH_4_Ac, 163.49 mS cm^−1^ for 1 M NH_4_Cl, and 143.54 mS cm^−1^ for 0.5 M (NH_4_)_2_SO_4_, as shown in Fig. S6 and Table S1. The pH values of the electrolytes were measured in Fig. S7, where 1 M NH_4_Ac was nearly neutral (pH = 6.9), whereas 1 M NH_4_Cl (pH = 5.8) and 0.5 M (NH_4_)_2_SO_4_ (pH = 5.4) were weakly acidic. In acidic media, H^+^ competes with NH_4_^+^ for intercalation. Owing to its smaller ionic radius, H^+^ can co-intercalate with NH_4_^+^ during insertion but preferentially deintercalates during extraction, thereby diminishing the reversible capacity.^26^ This accounts for the inferior cycling stability of HMND in acidic electrolytes. This indicates that the performance differences are not governed primarily by ionic conductivity, but rather by the pH effect. Beyond the pH effect, specific anion–electrode interactions also play a crucial role; Ac^−^ enables the formation of a coordination complex, which facilitates NH_4_^+^ desolvation and direct hydrogen bonding with the active sites, thereby enhancing the electrochemical kinetics. In contrast, SO_4_^2−^ and Cl^−^ do not exhibit this specific effect. Furthermore, the capacity derived from different scan rates (Fig. S5i) corroborates the superior electrochemical performance of the NH_4_Ac system.

**Fig. 2:**
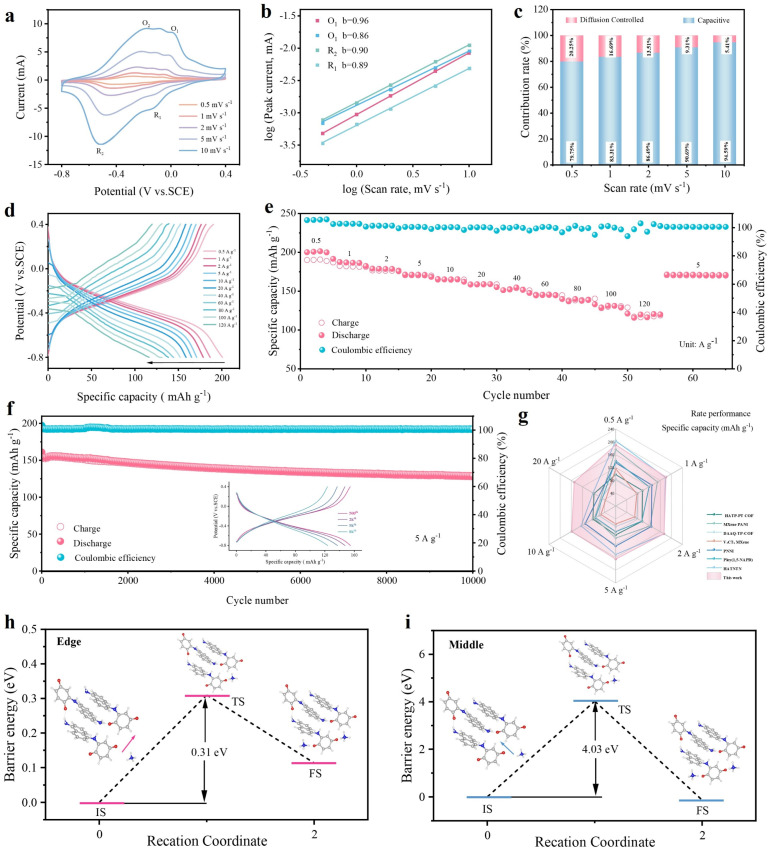
Electrochemical characterization of the HMND electrode. (a) CV curves at 0.5–10 mV s^−1^. (b) *b* values calculated corresponding to CV curves at different scan rates. (c) Capacitive contributions. (d) GCD curves of HMND at different current densities from 0.5 A g^−1^ to 120 A g^−1^. (e) Rate performance of HMND. (f) Cycling performance of HMND at a current density of 5 A g^−1^. (g) Rate performance comparison of HMND with other reported anode materials. (h) Energy barriers of NH_4_^+^ migration along molecular edges and perpendicular to layers. (i) Energy barriers of middle-interlayer NH_4_^+^ migration.

To optimize the electrochemical performance, HMND was evaluated in NH_4_Ac electrolytes with varying concentrations (1 M, 2 M, 5 M, 10 M, and 19 M). The results (Fig. S8 and S9) indicated that the HMND anode delivered its best overall performance at 5 M NH_4_Ac. As shown in Fig. S8c, the HMND electrode exhibited excellent rate capability across a wide current density range from 0.5 to 80 A g^−1^ in the 5 M NH_4_Ac electrolyte. The CV profiles maintained nearly identical shapes across different rates (Fig. S10 and 2a), demonstrating excellent electrochemical reversibility. Kinetic analysis yielded *b*-values close to 1 (Fig. 2b), suggesting that the redox reactions are predominantly governed by a surface-controlled process.^27^ Capacitive contribution analysis (Fig. 2c) further revealed that the proportion of surface-controlled capacity increased from 79.75% to 94.59% as the scan rate was increased from 0.5 to 10 mV s^−1^. The near-unity *b*-values and dominant capacitive contribution confirm that charge storage in HMND is primarily governed by fast NH_4_^+^ transport within the amorphous framework,^28^ providing the kinetic basis for its outstanding rate capability.

The exceptional kinetic properties of HMND are directly manifested in its galvanostatic charge–discharge (GCD) performance across a wide range of current densities. As depicted in Fig. 2d, the HMND electrode delivered a high discharge capacity of 200.7 mAh g^−1^ at 0.5 A g^−1^. Notably, even at an ultrahigh current density of 120 A g^−1^, it retained a capacity of 116.3 mAh g^−1^, with each full charge–discharge cycle requiring only ∼6.15 s, highlighting its efficient charge-storage capability under high-rate conditions. This remarkable rate performance is further evidenced by the capacity recovery test shown in Fig. 2e, where the capacity largely recovered upon returning to 5 A g^−1^ after cycling at 120 A g^−1^, revealing highly stable and reversible charge-storage behavior. Overall, the HMND electrode exhibited superior rate capability, which surpasses that of most reported organic electrode materials for aqueous ammonium-ion batteries (Fig. 2g and Table S2).^8,17,21,26,29–31^ The rapid charge-storage kinetics were corroborated by the galvanostatic intermittent titration technique (GITT) and temperature-dependent electrochemical impedance spectroscopy (EIS). GITT measurements (Fig. S11) revealed that the NH_4_^+^ diffusion coefficient in HMND during discharge ranged from approximately 10^−8^ to 10^−12^ cm^2^ s^−1^, higher than that of many comparable materials, indicating fast NH_4_^+^ migration within its amorphous framework.^32,33^ Furthermore, the activation energy for the interfacial reaction (Fig. S12), derived from fitting the Arrhenius equation, was calculated to be as low as 10.83 kJ mol^−1^. Such a low activation energy signifies an extremely small energy barrier for the NH_4_^+^ redox reaction at the electrode/electrolyte interface.^34^ The synergistic effect of this high bulk-phase diffusion rate and low-resistance interfacial reaction collectively endows the HMND anode with its outstanding rate capability at high current densities. These superior kinetics stem from the unique NH_4_^+^ transport mechanism in HMND. Furthermore, as shown in Fig. S13, the radial distribution function (RDF) analysis revealed a peak at approximately 2.5 Å for the H(NH_4_^+^)–C=O interaction and a peak at approximately 1.79 Å for the H(H_2_O)–C=O interaction, indicating the presence of hydrogen-bonding interactions between HMND and the electrolyte.^35^

Fig. 2h and i indicate that NH_4_^+^ migration along the molecular periphery encounters a significantly lower energy barrier compared to conventional intercalation through layered spaces. This edge-mediated non-intercalative transport pathway minimizes structural distortion and prevents the collapse of the crystal framework, thereby ensuring rapid and highly reversible ion storage even under a high current density. Fig. 2f displays the cycling performance of the HMND electrode at 5 A g^−1^. After 10 000 cycles, it manifested a stable cycling performance with a capacity retention of 82.81%, retaining a discharge specific capacity of 124 mAh g^−1^. Moreover, the coulombic efficiency (CE) remained at 100% without fluctuation, revealing the high reversibility and suppressed side reaction. Morphological and compositional analyses of the HMND electrode before and after cycling (Fig. S14) revealed that the overall electrode morphology was well preserved, with no significant particle pulverization or agglomeration. The corresponding elemental mapping further confirmed the uniform distribution of framework elements such as C, N, and O after cycling, indicating no loss of active components. Notably, even at an ultra-high current density of 120 A g^−1^, the HMND electrode delivers an exceptional capacity retention of about 90% after 30 000 cycles (Fig. S15). Collectively, these results demonstrated that HMND maintained excellent compositional and structural integrity during prolonged cycling, and the edge-mediated NH_4_^+^ transport pathway effectively preserves the original electrode morphology, which underpins its ultra-long cycle life.

**Fig. 3:**
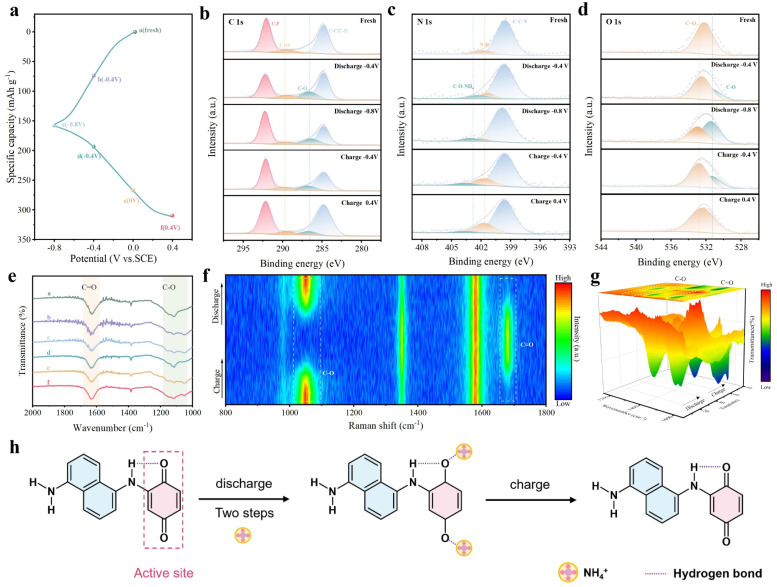
Charge–discharge mechanism of HMND. (a) *Ex situ* FT-IR spectra and *ex situ* XPS spectra of HMND corresponding to the points at different potentials. *Ex situ* XPS spectra of HMND: (b) C 1s, (c) N 1s, and (d) O 1s. (e) *Ex situ* FT-IR spectrum of HMND. (f) *In situ* Raman spectrum of HMND. (g) *In situ* FT-IR spectrum of HMND. (h) Schematic illustration of the possible coordination products of HMND with NH_4_^+^.

To further elucidate the NH_4_^+^ storage mechanism in HMND, a series of *ex situ* XPS and FT-IR spectroscopic characterization studies were conducted at different discharge/charge states (Fig. 3a). In the C 1s XPS spectra (Fig. 3b), the intensity of the C=O peak (289.3 eV) decreased during discharge compared to the fresh state, while the intensity of the C–O peak (287.9 eV) increased. This observation indicates the reduction of C=O to C–O upon NH_4_^+^ insertion. Correspondingly, the N 1s spectra (Fig. 3c) exhibited the emergence of a new component at 402.4 eV during discharge, which was attributed to nitrogen species involved in N–H⋯O hydrogen bonding, providing direct evidence for the coordination between NH_4_^+^ and the carbonyl oxygen.^11^ Upon charging, this diminished signal also confirmed the reversible dissociation of the C–O⋯NH_4_^+^ coordination structure and the re-oxidation of C–O back to C=O. The reversible transformation between C=O and C–O was further corroborated by the O 1s spectra (Fig. 3d), where the intensity of the peak corresponding to C=O (532.5 eV) weakened during discharge and recovered upon charging. *Ex situ* FT-IR analysis (Fig. 3e) tracked the evolution of functional groups at the molecular vibration level. As the discharge proceeded, the characteristic stretching vibration band of C=O at 1672 cm^−1^ gradually attenuated, while the band associated with C–O at 1160 cm^−1^ intensified. These reversible spectral changes collectively demonstrate the reduction of C=O to C–O and the concomitant formation of C–O⋯NH_4_^+^ hydrogen-bonded complexes during NH_4_^+^ storage, and their reverse oxidation during NH_4_^+^ release. Therefore, the combined *ex situ* spectroscopic evidence preliminarily reveals an NH_4_^+^ storage mechanism for HMND governed by the reversible redox reaction of the C=O/C–O couple coupled with hydrogen bonding.

To monitor the real-time evolution of C=O and C–O bonds during NH_4_^+^ (de)intercalation and thereby elucidate the reaction kinetics and reversibility, it is essential to perform *in situ* Raman and FT-IR spectroscopic studies. The *in situ* Raman spectrum (Fig. 3f) shows that, during discharge (NH_4_^+^ insertion), the intensity of the C=O stretching vibration (1670 cm^−1^ gradually decreases, while the peak associated with C–O (1080 cm^−1^) concomitantly increases. As shown in Fig. 3g, *in situ* FT-IR results further corroborate this transformation at the molecular vibration level. The highly reversible and perfectly synchronized responses of both *in situ* spectroscopic techniques directly confirm the real-time coupling between NH_4_^+^ (de)intercalation and the redox activity and reversibility of the C=O/C–O pair.

Based on the *ex situ* and *in situ* characterization studies described above, the NH_4_^+^ storage mechanism in HMND is proposed, as illustrated in Fig. 3h. Considering the presence of two C=O active sites with slightly different chemical environments in the HMND molecular structure, it is inferred that the NH_4_^+^ storage process involves two independent redox steps at different potentials,^36^ rather than a single reaction. During discharge, NH_4_^+^ ions migrate from the electrolyte and insert into the active sites of the HMND electrode, where they coordinate with the carbonyl oxygen atoms. This coordination leads to the reduction of the C=O bonds to C–O bonds, accompanied by the formation of C–O⋯NH_4_^+^ coordination structures. During charging, NH_4_^+^ ions are released from these structures back into the electrolyte, and the C–O bonds are oxidized back to C=O bonds, thereby restoring the original material framework. This highly reversible synergistic process, involving NH_4_^+^ coordination/dissociation coupled with the C=O/C–O redox reaction, constitutes the core mechanism, enabling efficient and stable ammonium-ion storage in HMND.^37^

To address the intrinsic limitations of manganese-based oxide cathodes for aqueous ammonium-ion batteries, including low conductivity, structural instability, and Jahn–Teller distortion,^38,39^ a series of carbon-coated MnO composites (MnO@C) were synthesized using polyvinyl alcohol (PVA) as the carbon source, with loadings of 0.5, 1.0, 1.5, and 2.0 g. Electrochemical performance (Fig. S16) revealed that an appropriate amount of PVA (≤1.5 g) promotes the formation of a conductive surface network while buffering volume strain, thereby enhancing both the electrical conductivity and structural stability, leading to improved capacity and cycling performance. Among the samples, MnO@C-1.5G exhibited the highest capacity retention and optimal structural stability. In contrast, excessive PVA (2.0 g) yielded an overly thick carbon layer after pyrolysis, which dilutes the active material and impedes bulk ion diffusion, resulting in capacity decay. Consequently, MnO@C-1.5G was selected as the cathode material for subsequent investigations.

**Fig. 4:**
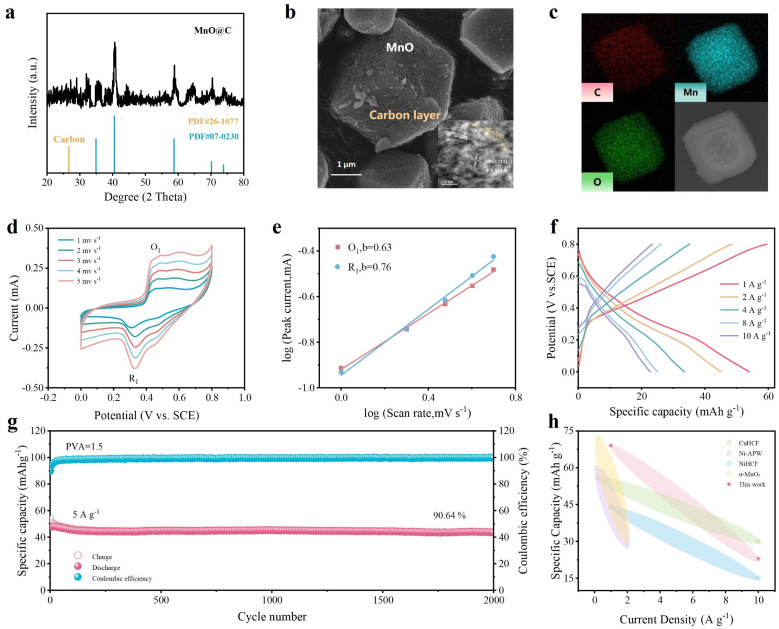
Morphology and electrochemical performance of the cathode. (a) XRD pattern. (b) SEM and HRTEM images. (c) EDS elemental mapping images. (d) CV curves at 1–5 mV s^−1^. (e) *b* value calculated from the corresponding CV curves at different scan rates. (f) GCD curves of MnO@C at different current densities from 1 A g^−1^ to 10 A g^−1^. (g) Cycling performance of MnO@C at a current density of 5 A g^−1^. (h) Rate performance comparison of MnO@C with other reported cathode materials.

X-ray diffraction (XRD) (Fig. 4a) confirmed that MnO@C retained the characteristic crystal structure of MnO without significant phase alteration induced by carbon coating. SEM/TEM images (Fig. 4b and S17) revealed a uniform cubic morphology, with the surface fully encapsulated by an ultrathin, continuous carbon layer (Fig. 4c). This core (MnO)–shell (C) architecture could facilitate efficient electron transport and suppress active material dissolution during cycling.^40–42^ Energy-dispersive X-ray spectroscopy (EDS) mapping (Fig. 4c) demonstrated a homogeneous distribution of C, O, and Mn, indicating effective integration between the carbon coating and the MnO matrix. High-resolution TEM imaging (Fig. 4b) showed lattice fringes with a spacing of 2.565 Å, corresponding to the (111) plane of MnO, which was further verified by selected-area electron diffraction (SAED) patterns (Fig. S18). TGA was employed to quantitatively determine the carbon content in MnO@C. As shown in Fig. S19, the TGA profile recorded under an oxygen atmosphere exhibited two distinct stages. A pronounced mass loss of approximately 6.25% occurred upon heating to about 296 °C, which was primarily attributed to the oxidative combustion of the amorphous carbon coating. Subsequently, a slight mass gain was observed above 300 °C, likely resulted from the further oxidation of the manganese oxide core to higher-valent oxides at elevated temperatures. (All specific capacities reported in this work were calculated based on the mass of the active material, excluding the carbon coating.)

CV curves (Fig. 4d) of the MnO@C electrode exhibit a pair of reversible redox peaks at 0.32/0.47 V (*vs.* SCE), corresponding to the Mn^2+^/Mn^3+^ redox couple. The linear fitting of the logarithmic relationship between the peak current and the scan rate yields *b*-values of 0.76 and 0.63 (Fig. 4e), indicating a hybrid charge storage mechanism involving both diffusion-controlled and pseudocapacitive processes. The quasi-rectangular shape of the CV curves further corroborates the dominant pseudocapacitive contribution, which provides the kinetic basis for its excellent rate capability.^43^ GCD measurements showed a discharge specific capacity of 58 mAh g^−1^ at 1 A g^−1^, with capacities of 47, 35, 26, and 24 mAh g^−1^ retained at 2, 4, 8, and 10 A g^−1^, respectively (Fig. 4f). When the current density was returned to 1 A g^−1^, the specific capacity recovered without significant decay (Fig. S20), demonstrating good rate performance and structural stability. Compared with previously reported cathode materials,^18,21,29,44^ the MnO@C electrode exhibited superior specific capacity across a wide range of current densities (Fig. 4h and Table S3). Long-term cycling tests revealed a capacity retention of 90.64% after 2000 cycles at 5 A g^−1^, with coulombic efficiency remaining close to 100% (Fig. 4g), which is primarily attributed to the protective effect of the carbon coating on the active material.^45^

To further elucidate the charge storage mechanism of MnO@C, *ex situ* X-ray photoelectron spectroscopy (XPS) analysis was performed on electrodes at different states of charge. The Mn 2p spectrum of the pristine electrode (Fig. S21) showed binding energies characteristic of Mn^2+^. During charging, the intensity of the Mn^2+^ peaks decreased significantly, while signals corresponding to Mn^3+^ increased markedly (Fig. S22), indicating the oxidation of Mn^2+^ to Mn^3+^ upon NH_4_^+^ extraction. This process was fully reversible upon discharge, with Mn^3+^ being reduced back to Mn^2+^ as NH_4_^+^ was re-inserted into the structure. These results demonstrate that the reversible Mn^2+^/Mn^3+^ redox couple contributed substantially to the overall capacity.

**Fig. 5:**
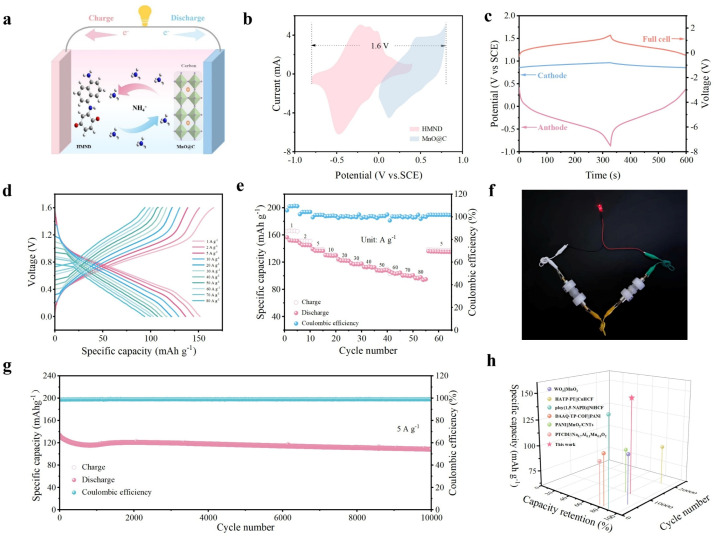
Electrochemical performances of HMND//MnO@C. (a) Illustration of the HMND//MnO full battery. (b) Potential distribution of the HMND anode and MnO@C cathode, as shown by CV curves. (c) Chronopotentiometry curves of the HMND//MnO@C full battery, HMND anode, and MnO cathode at a current density of 2 A g^−1^. (d) GCD curves of the full battery at different current densities from 1 A g^−1^ to 80 A g^−1^. (e) Rate performance of the full battery. (f) The digital photo of the LED light lit up by the full battery. (g) Cycling performance of the full battery at a current density of 5 A g^−1^. (h) A comparison of performance of this work with other reported work in AAIBs.

Based on the excellent electrochemical performance of the individual electrodes, a rocking-chair aqueous NH_4_^+^-ion battery was assembled using HMND as the anode, MnO@C as the cathode, and 5 M NH_4_Ac as the electrolyte, as illustrated in Fig. 5a. Initially, CV curves (Fig. 5b) showed the operating potential windows of the cathode and anode. It was found that the HMND anode exhibited a characteristic negative potential response within the range of −0.8 V to +0.4 V (*vs.* SCE), while the MnO@C cathode displayed a stable positive potential response from 0 V to +0.8 V (*vs.* SCE). Accordingly, a theoretical operating voltage window of up to 1.6 V was established for this battery system. Fig. 5c presents the GCD profiles (voltage *vs.* time) of the full battery, along with the corresponding potential evolution of the cathode and anode (potential *vs.* time). It can be clearly observed that the discharge plateau of the full battery originates from the sustained potential difference between the cathode (red line) and the anode (blue line), resulting in a stable operating voltage window of 0–1.6 V, and the minimal polarization further indicated well-matched reaction kinetics between the electrodes. Examination of Fig. 5d demonstrated that the battery can deliver a high specific capacity of 151.6 mAh g^−1^ at a current density of 1 A g^−1^ (calculated based on the anode mass). Even when subjected to an extremely high current density of 80 A g^−1^, a capacity of 93.8 mAh g^−1^ was retained, demonstrating exceptional rate capability. Upon returning the current density from 80 A g^−1^ to 5 A g^−1^, the specific capacity was nearly fully recovered (Fig. 5e), highlighting the excellent structural reversibility and kinetic stability of the materials. Its practical viability was visually demonstrated by successfully powering an LED lamp (Fig. 5f). Furthermore, the full battery exhibited outstanding long-term cycling stability. After 10 000 consecutive charge–discharge cycles at a current density of 5 A g^−1^, the specific capacity remained at 108 mAh g^−1^, corresponding to the high capacity retention of 81.63% (Fig. 5g). Importantly, the initial capacity decay during the first ∼50 cycles was attributed to electrode activation, a common phenomenon in organic electrodes, after which the capacity stabilized and remained highly stable over the subsequent cycles. At a lower current density of 1 A g^−1^, the full battery also delivered stable cycling performance, retaining 86.40% of its initial capacity after 1000 cycles (Fig. S23). Additionally, the self-discharge behavior of the full cell was evaluated (Fig. S24). After a 24 h open-circuit test, a significant self-discharge effect was observed. This substantial voltage loss was most likely attributable to dissolved oxygen within the aqueous electrolyte system.^46,47^ A comparison of these performance metrics (Fig. 5h and Table S4), including maximum specific capacity, cycle life, and capacity retention, with those of previously reported ammonium-ion full batteries further highlights its superior overall performance.^17,28,29,48–50^

**Fig. 6:**
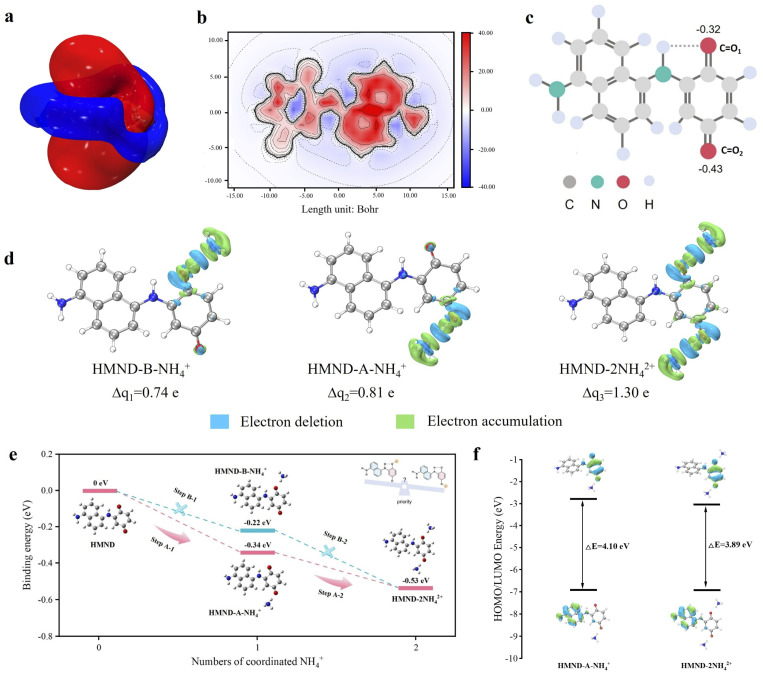
Theoretical calculations of NH_4_^+^ storage mechanisms. (a) Isosurface of ICSSZZ. (b) Color-filled contour map of ICSSZZ on the *XY* cross-section perpendicular to the symmetry plane of the HMND molecule. (c) Charge population sum of carbonyl head. (d) Charge density difference isosurfaces for different coordination states. (e) Binding energy of the optimized geometry. (f) Frontier molecular orbital diagrams and energy levels of HMND in different states.

The IsoChemical Shielding Surface (ICSS) technique was employed to investigate the electronic structure and aromaticity of the HMND molecule. Fig. 6a displays the Z-component of the ICSS (ICSSZZ) for the symmetric supramolecular plane, revealing a prominent shielded region (indicated in red) perpendicular to the plane, surrounded by a closed deshielding isosurface (shown in blue). This ICSSZZ distribution confirms the π-aromaticity of HMND. Two-dimensional cross-sections along the *XY* and *XZ* planes (Fig. 6b and S25) further illustrate the ICSSZZ distribution. These cross-sections clearly show that the shielded and deshielded regions are distinctly separated by red and blue colors, with only the blue area encircled by red. The red region occupied most of the HMND molecular area, exhibiting the strongest magnetic shielding, particularly within the quinone-based groups. It is speculated that the carbonyl-rich framework facilitates complete π-electron delocalization. Therefore, the delocalization of π-electrons across the conjugated superstructure promotes electron injection into the HMND molecule with a low energy barrier, thereby enhancing structural stability.^20^

Furthermore, the charge populations of the two carbonyl groups in HMND were −0.32 and −0.43 a.u., respectively (Fig. 6c). The C=O_2_ group exhibits a more negative charge, suggesting a stronger coupling capability and a higher affinity for forming coordination complexes with charge carriers.^51^Fig. 6d displays the charge density differences for three coordination modes, with values of 0.74*e*, 0.81*e*, and 1.30*e*. Since the C=O groups in HMND act as electron donors, the reduction in their absolute charge upon NH_4_^+^ coordination corresponds to enhanced electron transfer, which is favorable for NH_4_^+^ storage. Therefore, the coordination mode with the largest charge density difference corresponds to the maximum electron transfer. To further confirm the preferred attack direction of NH_4_^+^, the binding energies for two possible pathways were calculated (Fig. 6e). In Path A-1, the NH_4_^+^ ion attacks the C=O_2_ group to form an N–H⋯O hydrogen-bond, generating an intermediate labeled HMND-A-NH_4_^+^. In Path B-1, NH_4_^+^ preferentially coordinates with the other carbonyl group C=O_1_, accompanied by a one-electron transfer, yielding the intermediate HMND-B-NH_4_^+^. The calculated binding energy for forming HMND-A-NH_4_^+^ was −0.34 eV, which was significantly lower than that for HMND-B-NH_4_^+^ (−0.22 eV). According to the energy minimization principle, the coordination configuration HMND-A-NH_4_^+^ generated *via* Path A-1 is thermodynamically more stable. The second discharge step also involves a one-electron transfer, as shown in Path A-2, where NH_4_^+^ coordinates with the remaining C=O_1_ group to form the final product HMND-2NH_4_^2+^.

To elucidate the evolution of the electronic structure of HMND during energy storage, the frontier molecular orbitals of its different charged states (HMND-A-NH_4_^+^ and HMND-2NH_4_^2+^) were calculated (Fig. 6f). The calculations reveal that the LUMO energy levels of the intermediate state HMND-A-NH_4_^+^ and the final state HMND-2NH_4_^2+^ were as low as −2.77 eV and −3.02 eV, respectively, with corresponding HOMO–LUMO gaps of 4.10 eV and 3.89 eV, respectively. According to frontier molecular orbital theory, the process of a redox-active material accepting electrons corresponds to the filling of its LUMO.^52^ A lower LUMO energy level indicates higher electron affinity and stronger electron-accepting ability, thereby rendering the material more easily reduced. The continuous decrease in the LUMO energy level upon progressive NH_4_^+^ insertion indicates a corresponding continuous increase in the electron affinity of HMND, confirming its role as an efficient active site for NH_4_^+^ storage. Furthermore, the electrostatic potential (ESP) map of HMND reveals that its carbonyl regions reside in more negative ESP value zones,^20^ further indicating that HMND maintains strong electron-capturing capability throughout the multi-step NH_4_^+^ storage process. The single-molecule DFT model captures the local redox behavior of HMND and is justified by the edge-mediated non-intercalative NH_4_^+^ transport mechanism.

## Conclusions

In summary, this work presents the successful construction of a novel organic polymer anode HMND, by integrating the benzoquinone active unit with a hydrogen-bond network design strategy. This material architecture not only effectively suppresses electrode dissolution and enhances structural integrity but also, in synergy with the extended π-conjugated framework, facilitates efficient electron delocalization. Crucially, the hydrogen-bonding network orchestrates a unique NH_4_^+^ transport pathway. This non-intercalative mechanism enables NH_4_^+^ ions to migrate along the molecular periphery with low energy barriers, minimizing structural distortion and ensuring rapid kinetics. As a result, the HMND anode delivers a capacity of 116.3 mAh g^−1^ even at an ultra-high current density of 120 A g^−1^ and maintains a high capacity retention of 82.81% after 10 000 cycles at 5 A g^−1^. The corresponding HMND//MnO@C full battery also demonstrates outstanding NH_4_^+^ storage performance, achieving a high capacity of 151.6 mAh g^−1^ at 1 A g^−1^ and retaining a stable capacity of 108 mAh g^−1^ after 10 000 cycles at 5 A g^−1^. Combined theoretical calculations and spectroscopic characterization reveal that HMND stores energy reversibly *via* a two-step coordination mechanism, facilitated by the reversible hydrogen bonds formed between NH_4_^+^ and its carbonyl sites. This work through intramolecular hydrogen-bond engineering and rational electrode material design provides an innovative and effective strategy for developing high-capacity, long-life AAIBs.

## Author contributions

Xingru Chen conducted the experiments and wrote the initial draft; Rong Ge, Xueqing Ren, Wei Qin and Yu Ge performed the theoretical calculations; Junyue Luo, Guangzheng Xu, and Jiamin Zhang carried out the formal analysis; Yongzheng Zhang, Duan Bin, Beibei Yang, and Hongbin Lu were responsible for the conceptualization, reviewing and editing of the article, as well as securing the research funding; Yonggang Wang assumed the supervisory work, reviewed and edited the article.

## Conflicts of interest

There are no conflicts to declare.

## Supplementary Material

SC-OLF-D6SC04614B-s001

## Data Availability

The data can be provided upon request. The data supporting this article have been included as part of the supplementary information (SI). Supplementary information is available. See DOI: https://doi.org/10.1039/d6sc04614b.
