# Supplementary material for: A hydrogen-bonded conjugated quinone polymer enables ultrafast and ultrastable NH4+ storage for aqueous ammonium-ion batteries
Source: Chem Sci. 2026 Jul 17;17(35):16807–17. doi: 10.1039/d6sc04614b (PMC13392992; doi:10.1039/d6sc04614b)
Supplement: Supplementary file 1 [file SC-017-D6SC04614B-s001.pdf]

Supplementary Information (SI) for Chemical Science.
This journal is © The Royal Society of Chemistry 2026

Supplementary Information

A Hydrogen-Bonded Conjugated Quinone Polymer Enables Ultrafast and Ultra-Stable NH_4^+ Storage for Aqueous Ammonium-Ion Batteries

Xingru Chen[a], Rong Ge[a], Xueqing Ren[a], Wei Qin[a], Yu Ge[a], Junyue Luo[a], Guangzheng Xu[a], Jiamin Zhang[a], Beibei Yang[a], Yongzheng Zhang[b], Duan Bin[a], Hongbin Lu[b*] and Yonggang Wang[c*]*

[a] Department of Polymer Materials and Science, College of Chemistry and Chemical Engineering, Nantong University, Nantong, 226019, China

[b] Haian Institute of High-end Textile, Nantong University, Nantong, 226000, P R China.

[C] Department of Chemistry and Shanghai Key Laboratory of Molecular Catalysis and Innovative Materials, Institute of New Energy, iChEM (Collaborative Innovation Center of Chemistry for Energy Materials), Fudan University, Shanghai 200433, China.

* E-mail: dbin17@fudan.edu.cn, luhb@nju.edu.cn, ygwang@fudan.edu.cn

Experimental Section

Reagents and Materials

1,5-Diaminonaphthaslene (1,5-NAD) was purchased from Shanghai Aladdin Biochemical Technology Co. Ltd. p-Benzoquinone (BQ) was purchased from Shanghai Macklin Biochemical Technology Co. Ltd. Ethanol absolute was purchased from Shanghai Rich Joint Technology Co. Ltd. Ethyl acetate was purchased from Xilong Scientific Co. Ltd. Potassium permanganate was purchased from Shanghai Lingfeng Chemical Reagent Co. Ltd. Polyvinyl alcohol was purchased from Shanghai Titan Technology Co. Ltd. Manganous chloride tetrahydrate was purchased from Shanghai Aladdin Biochemical Technology Co. Ltd. Polytetrafluoroethylene preparation, 60% (PTFE) was purchased from Beijing Inno Chem Science & Technology Co. Ltd. The deionized water used in this work was from a Millipore System.

Synthesis of HMND and MnO@C

HMND Anode

1,5-NAD (0.3164g) and BQ (1.081g) was added into solvent that composed by 50 mL of ethanol, and then mixture was heated and stirred at 70 ℃ for 5 h. After cooling to room temperature, the precipitate was collected by centrifugal precipitation and washed successively with ethanol and ethyl acetate. Finally, HMND was dried overnight in a vacuum oven at 70 ℃. All reagents were used without further purification. The yield of HMND is approximately 35–40% after washing and drying.

MnO@C Cathode

1.5 g of polyvinyl alcohol (PVA) was dissolved in 30 mL of deionized (DI) water under heating and stirring at 90 °C for one hour. The resulting hot PVA solution was then added dropwise into 30 mL of a 0.125 mol L^{-1} potassium permanganate ($KMnO_4$) solution. After magnetically stirring the mixture for 30 minutes, 10 mL of a 0.125 mol L^{-1} manganese (II) chloride tetrahydrate ($MnCl_2 \cdot 4H_2O$) solution was introduced, followed by an additional 30 minutes of stirring. The mixture was subsequently

transferred to a Teflon-lined stainless-steel autoclave and reacted at 180 °C for 30 minutes. Upon completion of the reaction, the precursor was cooled to room temperature, rinsed with DI water, and dried at 60 °C. The resulting material was then calcined at 500 °C for 2 hours under a nitrogen atmosphere, with a heating rate of 5 °C/min. The final powder sample was designated as MnO@C-1.5G. For comparison, control samples were prepared under the same experimental conditions but with varying amounts of PVA (0, 0.5, 1.0, and 2.0 g), corresponding to MnO, MnO@C-0.5G, MnO@C-1G, and MnO@C-2G, respectively.

Materials Characterization

The morphology and microstructure of the samples were characterized using scanning electron microscopy (Gemini 300) and transmission electron microscopy (ThermoFisher Scientific Talos F200x G2 STEM). X-ray diffraction patterns were collected on a Bruker D8 Endeavor X-ray diffractometer (Cu Kα, λ = 0.15405 nm) over a 2θ range of 2–40° at a scan rate of $1^{\circ}\cdot min^{-1}$. X-ray photoelectron spectroscopy measurements were performed on a XSAM800 Ultra spectrometer (PHI 5000C & PHI 5300) to analyze the surface elemental composition and valence states of the materials, with the C 1s peak at 284.8 eV used as the reference for energy calibration. Fourier transform infrared spectra were acquired on a Thermo-Fisher Nicolet 6700 spectrometer to detect functional group signals within the specified wavenumber range. Solid-state nuclear magnetic resonance experiments were carried out on a Bruker 400M NMR spectrometer. Thermogravimetric analysis was conducted using a Rigaku thermal analyzer.

Electrode Preparation

The HMND electrode (anode) was prepared as follows: the synthesized HMND powder, KB conductive agent, and PTFE binder were blended at a mass ratio of 6:3:1 in isopropanol, after which the mixture was rolled into a uniform film using a roller machine. After drying at 60 °C for 12 h in a vacuum oven, the electrode film containing

HMND active material (with a loading of 2 mg·cm^{-2}) was pressed onto a titanium mesh current collector under a pressure of 30 MPa. The MnO@C electrode (cathode) was fabricated from MnO@C-1.5G powder, KB conductive agent, and PTFE at a mass ratio of 6:3:1, following the same preparation procedure and active material loading as the HMND electrode. The counter electrode, composed of active carbon (AC), KB, and PTFE at a mass ratio of 7:2:1, was prepared similarly to the main electrodes. The active carbon film was then pressed onto the titanium mesh with a mass loading of 15 mg·cm^{-2}.

Electrochemical Measurements

Electrochemical analyses of the cathode and anode materials were performed using a conventional three-electrode system: An AC electrode served as the counter electrode, a $Hg/HgCl_2$ electrode as the reference electrode, and the anode (or cathode) material as the working electrode, tested in different electrolytes. Cyclic voltammetry (CV) and galvanostatic charge–discharge (GCD) measurements were carried out at various scan rates and current densities, while electrochemical impedance spectroscopy (EIS) was conducted at open-circuit potential over a frequency range from 0.1 Hz to 10^5 Hz with a sinusoidal voltage amplitude of 10 mV. Additionally, the electrochemical behaviors of the HMND anode, MnO@C-1.5G cathode, and the full cell in 5 M NH_4Ac electrolyte were investigated via CV and GCD methods. All the above electrochemical measurements were performed on a Bio-Logic SP-200 electrochemical workstation and a Land CT 3002A multi-channel battery testing system. All specific capacities reported in this work are calculated and presented based solely on the mass of the active material.

Full-cell Assembly

A pretreatment step was performed on the MnO@C cathode prior to full-cell assembly. Specifically, the MnO@C cathode was first subjected to galvanostatic charge/discharge (GCD) cycling at a current density of 2 A g^{-1} in a half cell configuration

until the Coulombic efficiency (CE) stabilized. After the CE was stable, the pretreated cathode was removed and assembled into the full cell with the HMND anode. (N/P=1.3:1)

The HMND//MnO@C rocking-chair aqueous ammonium-ion full battery was assembled using a Swagelok-type two-electrode corrosion-resistant cell. The HMND electrode was used as the anode, and the MnO@C electrode was used as the cathode. A glass fiber separator was placed between the two electrodes, and 5 M NH_4Ac was used as the electrolyte. The cell was assembled in a standard Swagelok configuration, ensuring good contact between the electrodes and the current collectors.

Activation Energy

The activation energy (E_a, kJ mol^{-1}) for the charge transfer process can be derived from the Arrhenius equation.[1]

$$R_{ct}{}^{-1} = Aexp(-E_a/RT)$$

In this formula, $R_{ct}{}^{-1}$ represents charge transfer resistance (Ω), A is a constant under a stable condition, R is the gas constant (8.314 J mol^{-1} K^{-1}), and T is the temperature (K). The activation energy (E_a) was calculated based on the slope of the linear fit obtained from plotting ln(1/Rct) against 1000/T.

$$In(R_{ct}{}^{-1} = \frac{-E_a}{RT} + k)$$

Calculation of Capacitance Contribution

The charge storage kinetics of HMND were analyzed using CV curves measured at various scan rates, as described by the following relationship.[2]

$$i = av^b$$

where k and b are constants, i represents the current density, and v represents the scan rate. When the power exponent b value approaches 0.5, the reaction process is considered to be diffusion-controlled. Conversely, when b value is close to 1, it indicates a surface-controlled redox reaction process.

According to Dunn's method, the capacitive effects contributed by the surface-controlled and diffusion-controlled processes can be quantitatively analyzed using the following equation:[3]

$$i = k_1 v + k_2 v^{\frac{1}{2}}$$

where k_1 and k_2 are constants, with $k_1 v$ and $k_2 v^{\frac{1}{2}}$ representing the current density contributed by the fast-capacitive process and the diffusion-controlled process, respectively. Dividing both sides of the equation by $v^{\frac{1}{2}}$ gives:

$$i \,/\, v^{\frac{1}{2}} = k_1 v^{\frac{1}{2}} + k_2$$

Linear fitting reveals that the slope corresponds to k_1 and the intercept corresponds to k_2.

Galvanostatic Intermittent Titration Technique (GITT)

The NH_4^+ ion diffusion coefficients in the HMND electrode were calculated from the GITT data. In the GITT method, the transient voltage generated by applying a current pulse was monitored as a function of time. Prior to the GITT measurements, the assembled cell was first cycled 10 times at a current density of 0.2 A g^{-1} to achieve a stable state. Subsequently, a galvanostatic pulse with a current density of 0.2 A g^{-1} was applied for 10 minutes, followed by a 120-minute relaxation period to allow the system to return to equilibrium. The chemical diffusion coefficient was then obtained as follows:[4]

$$D_{GITT} = \frac{4}{\pi\tau}\left(\frac{m_B V_M}{M_B S}\right)^2\left(\frac{\Delta E_s}{\Delta E_\tau}\right)^2$$

Where ΔE_τ represents the potential change during the constant current pulse, while ΔE_s denotes the potential difference during the open-circuit period. D_{GITT} ($cm^2 \cdot s^{-1}$)

indicates the NH_4^+ diffusion rate, and m_B, M_B, V_M, τ, and S refer to the mass, molar mass, molar volume of the sample, duration of the constant current pulse, and electrode-electrolyte interface area, respectively.[5]

Density functional theory (DFT) calculations

All density functional theory (DFT) calculations were conducted using Gaussian16 software package, Revision C.01. Geometry optimization of all molecules was performed with M06-2X functional and the def2-SVP basis set. The DFT-D dispersion correction and the integral equation formalism variant of the polarizable continuum model were used in all calculations. The following single point calculations were performed with the same functional and the def2-TZVP basis set. The Atomic dipole corrected Hirshfeld atomic charge (ADCH)[6] was evaluated by Multiwfn 3.8(dev); the diagram of electron density difference and iso-chemical shielding surfaces (ICSS)[7] were also evaluated by Multiwfn. All Fig.s were rendered by Visual Molecular Dynamics (VMD) 1.9.3.

Spin-polarized first-principles computations were conducted within the framework of density functional theory (DFT) via the Vienna Ab initio Simulation Package (VASP)[8,9]. The calculations adopted the PBE-GGA functional[10] augmented with DFT-D3 dispersion corrections for van der Waals interactions. Electron-ion interactions were described by the projector augmented wave (PAW) method, utilizing a plane-wave energy cutoff of 450 eV, which was confirmed to ensure convergence within 1 meV/atom. Structural optimization was carried out with the following convergence thresholds: energy change below 10^{-5} eV between electronic iterations, atomic forces less than 0.01 eV/Å, and stress components under 0.02 kBar.

Molecular dynamics (MD) simulations were performed to investigate the structural characteristics. Initially, the geometry of the constructed model was optimized, followed by energy minimization using the Forcite module (10,000 steps) to obtain the lowest-energy configuration. Subsequently, MD simulations were conducted under the isothermal–isobaric (NPT) ensemble at 298 K for 100 ps to reach equilibrium, with

a time step of 1 fs and a pressure of 0.01 GPa. The final snapshot of the NPT run was then employed as the starting point for further equilibration under the canonical (NVT) ensemble for 400 ps. The COMPASS III force field was adopted throughout the simulations. Temperature and pressure were controlled using the Andersen and Berendsen thermostats/barostats, respectively.

Supplementary Figures

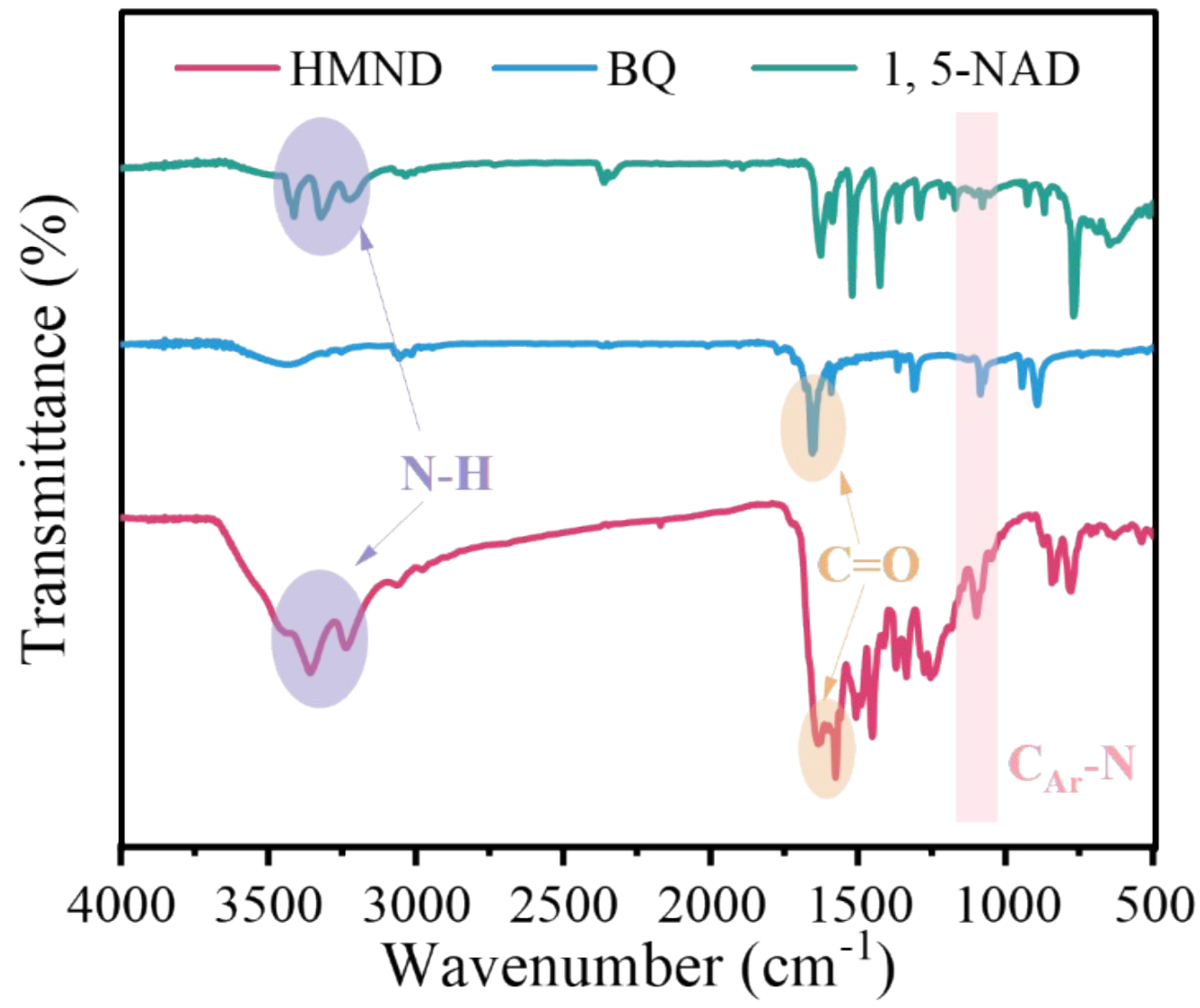

Fig. S1 FTIR spectrum of HMND, BQ and 1, 5-NAD.

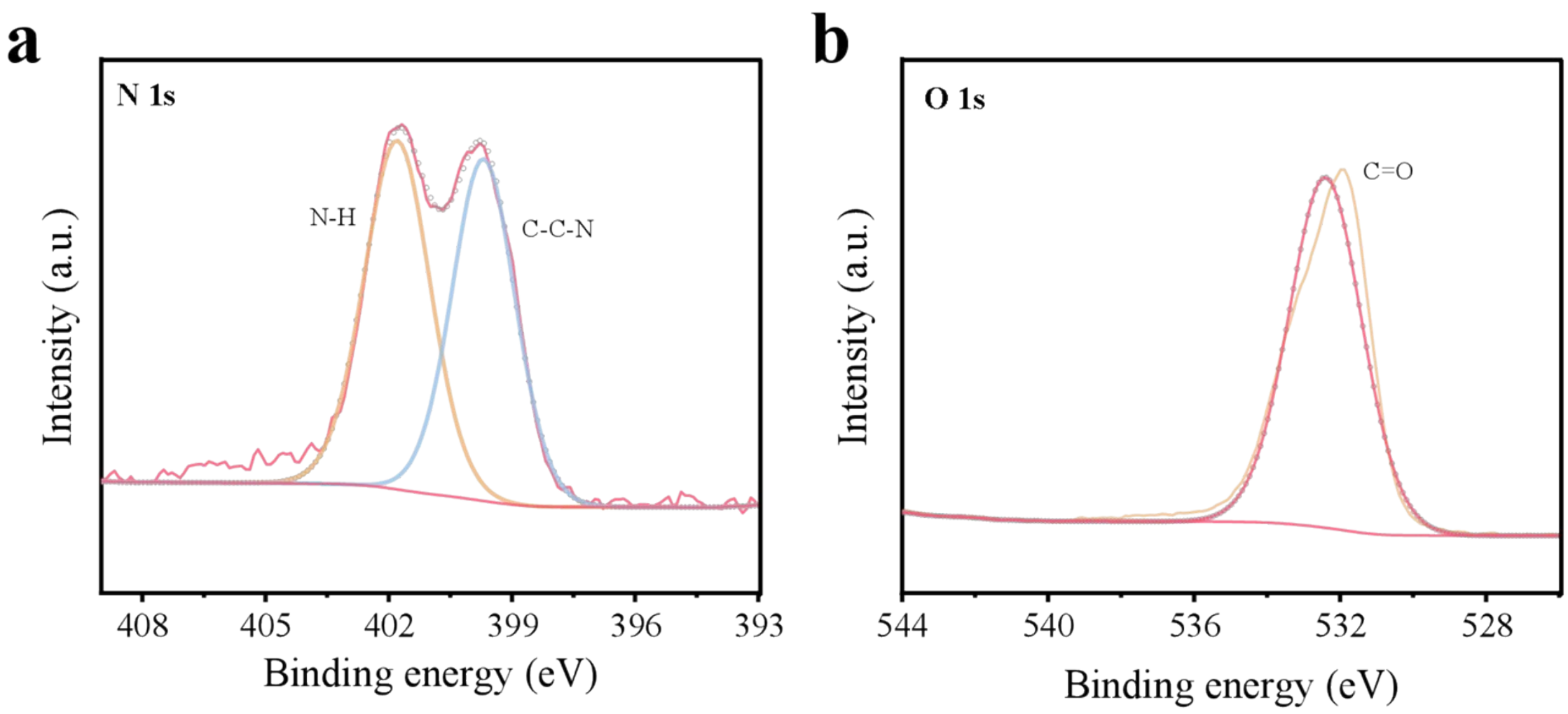

Fig. S2 High-resolution XPS spectra of HMND. (a) N 1s, (b) O 1s.

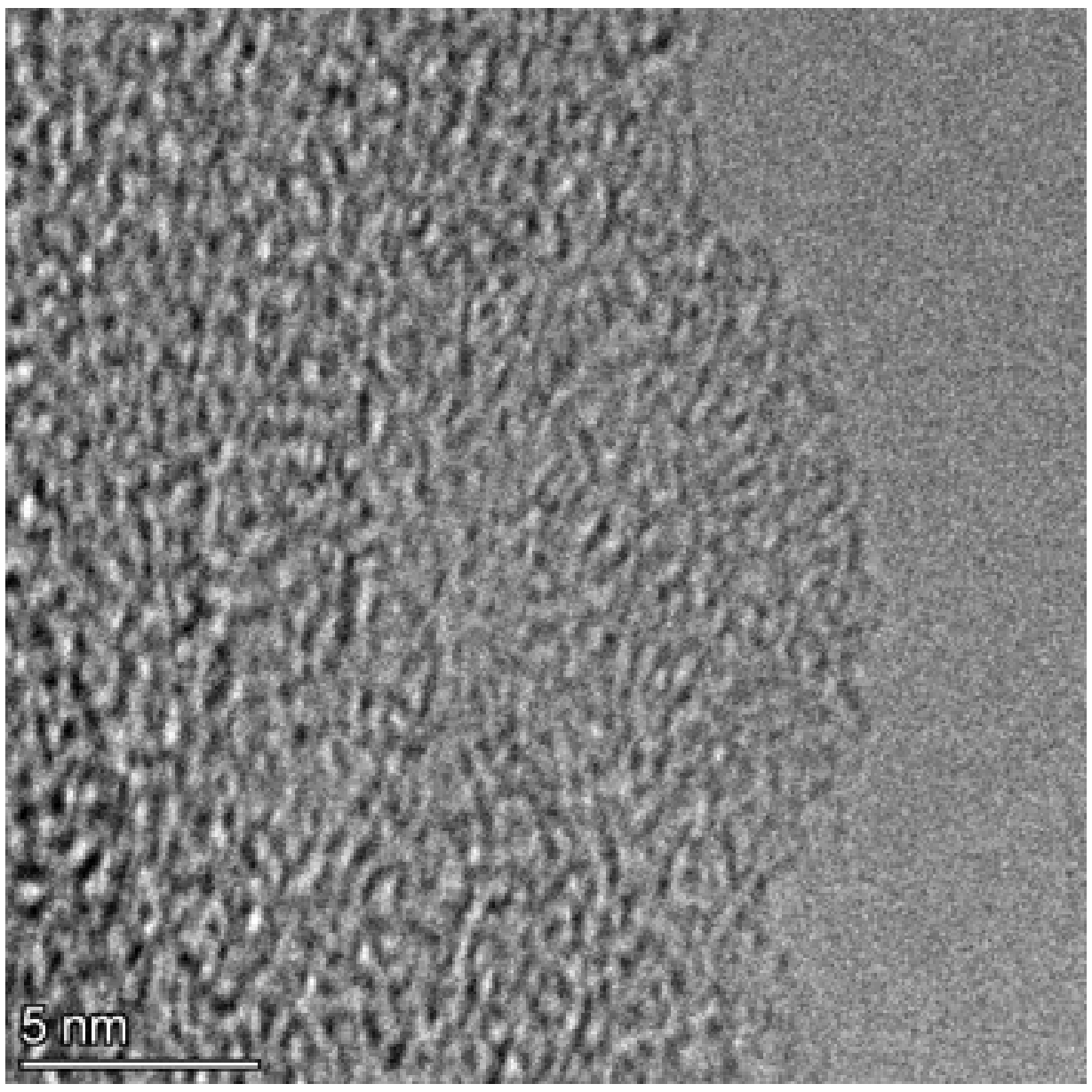

Fig. S3 HR-TEM image of HMND at 5 nm scale.

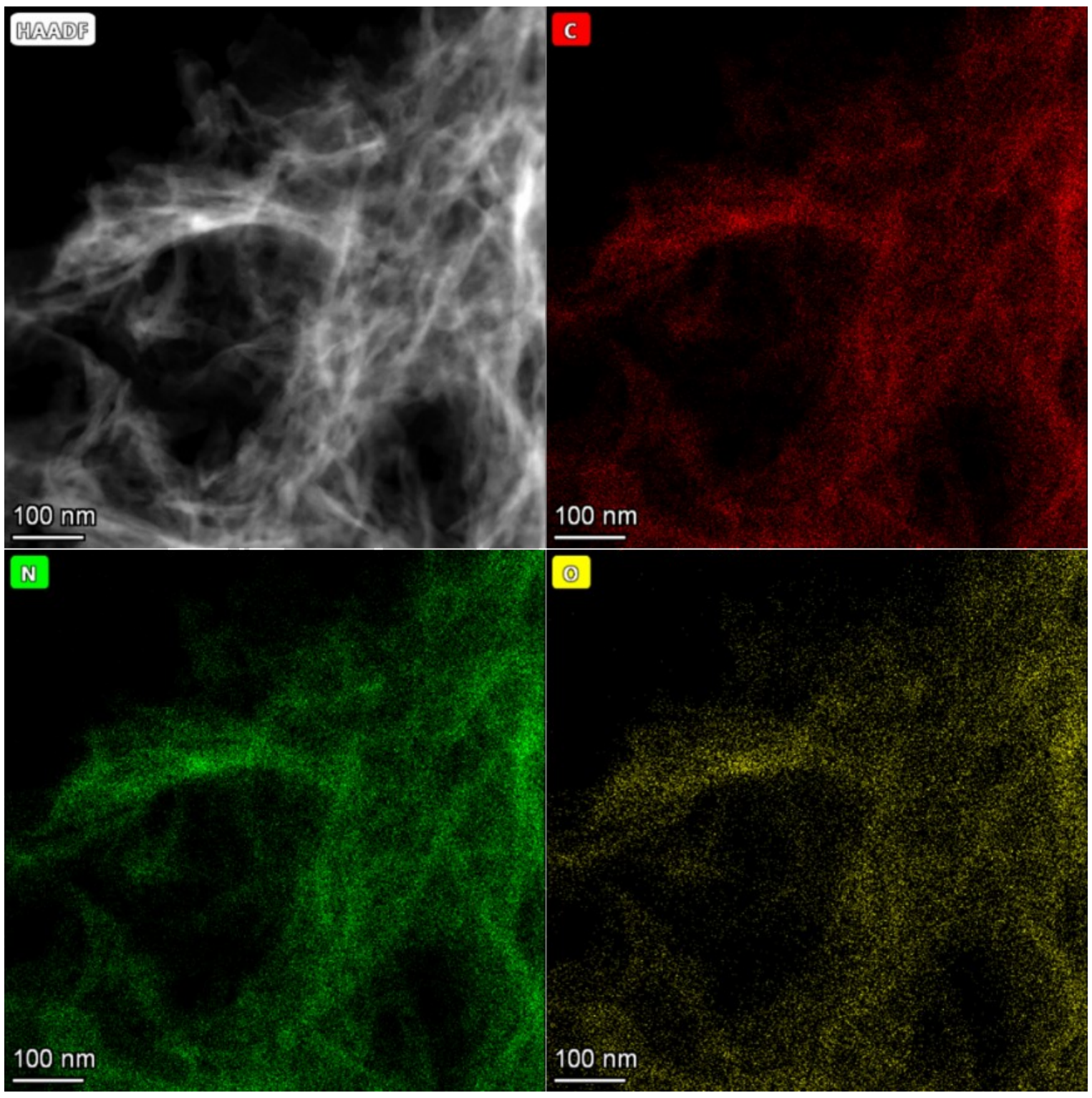

Fig. S4 EDS mappings of C, N and O atoms in HMND powder.

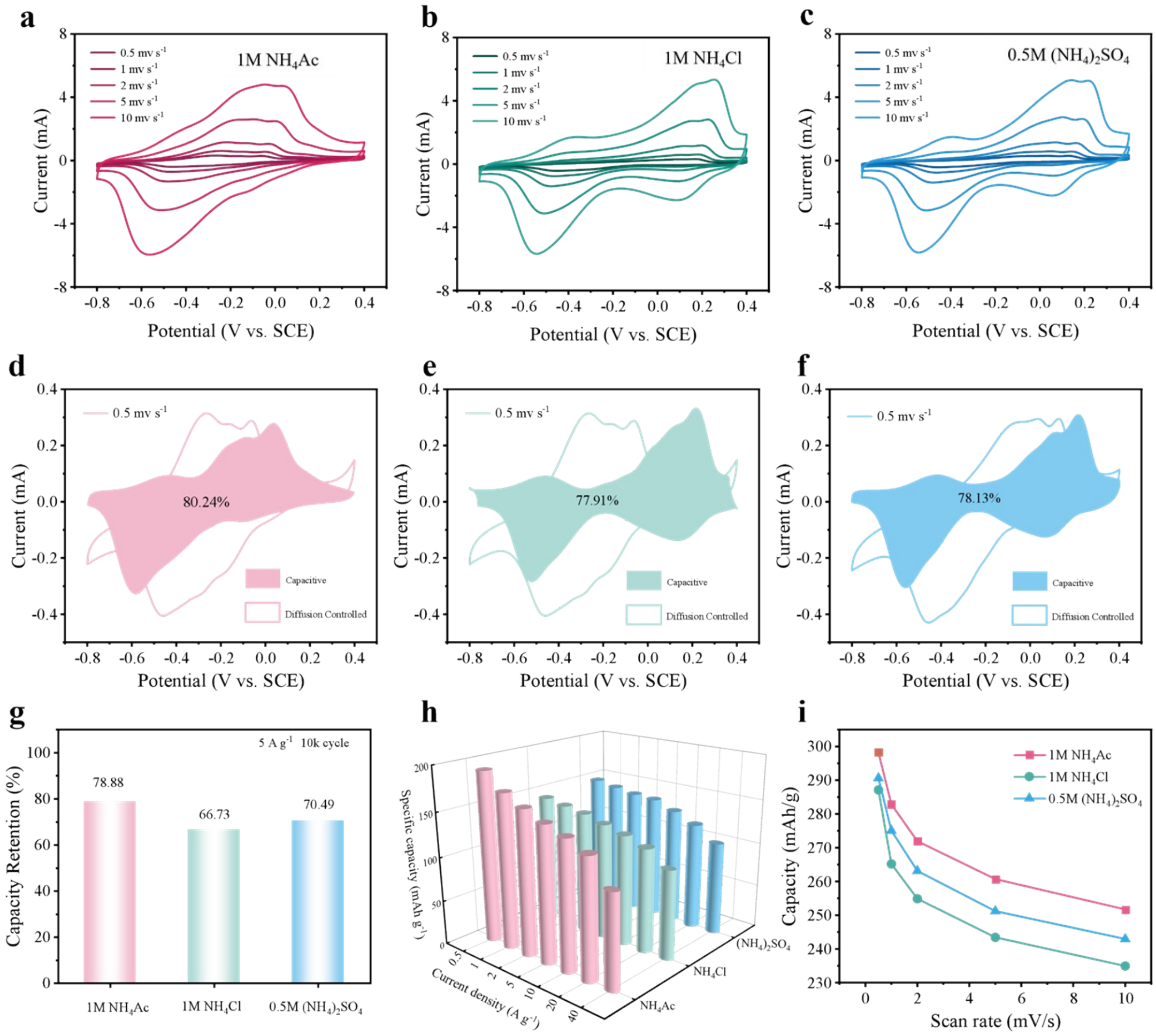

Fig. S5 (a−c) CV curves of HMND electrode with 1 M NH_4Ac, 1 M NH_4Cl , and 0.5 M $(NH_4)_2SO_4$ at different scan rates, (d−f) k_1 value at 0.5 mV s^{-1}, (g) Capacity retention in three kinds of electrolyte, (h) Rate performance and (i) capacity at different scan rates of HMND electrode in the different electrolyte.

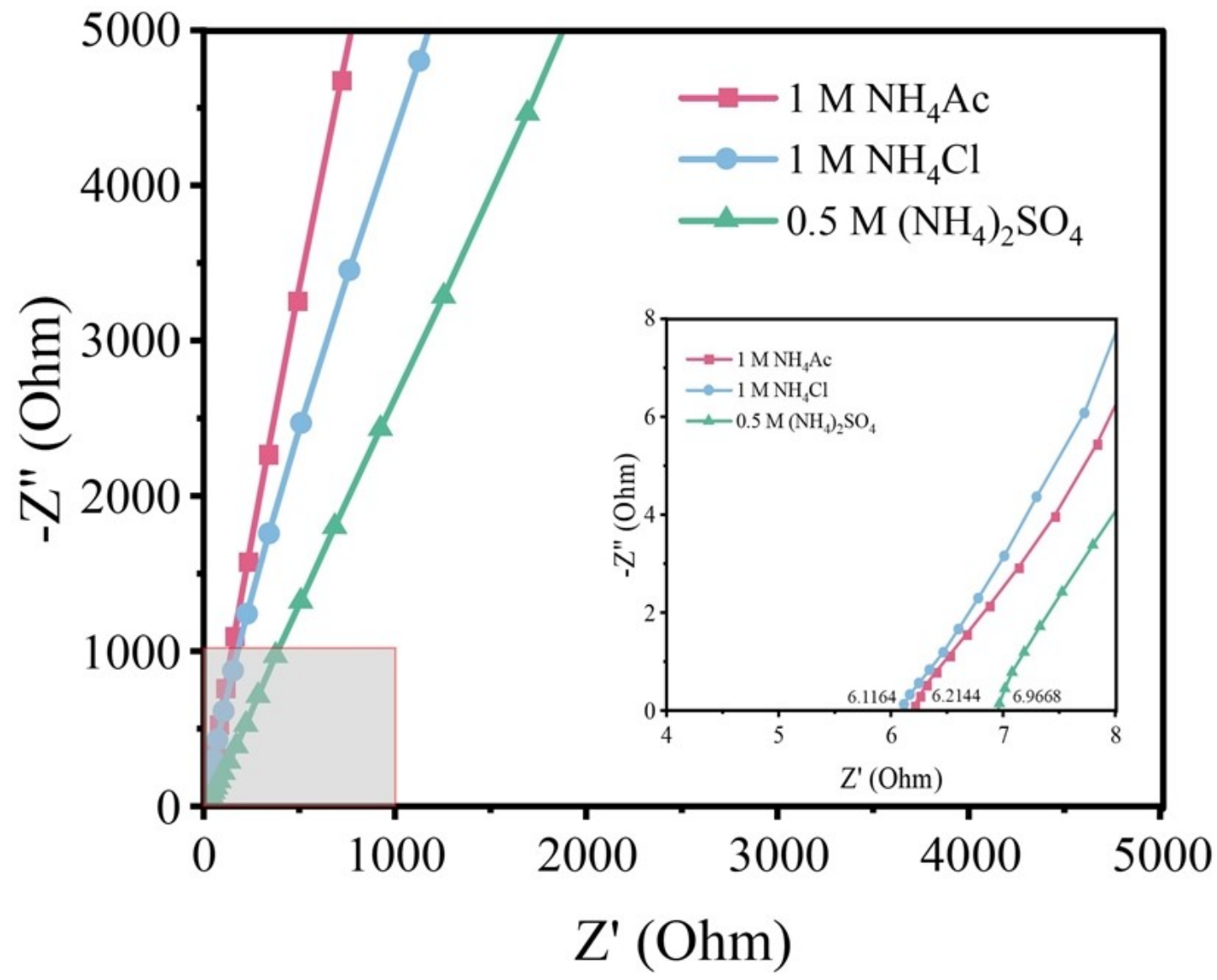

Fig. S6 Nyquist plots of the 1 M NH_4Ac, 1 M NH_4Cl, and 0.5 M $(NH_4)_2SO_4$ electrolytes measured at room temperature.

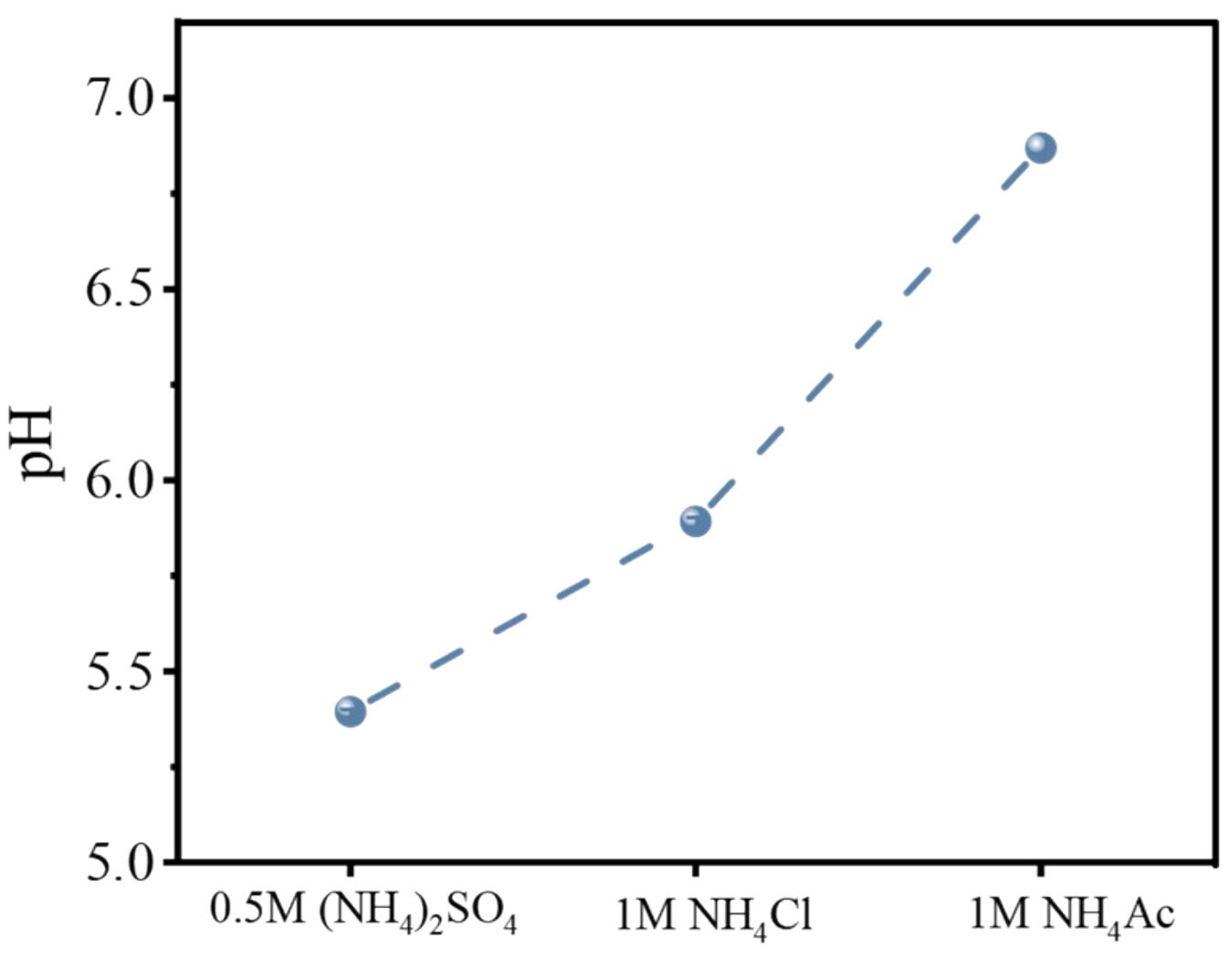

Fig. S7 pH values of 0.5 M $(NH_4)_2$ SO_4, 1 M NH_4Cl, and 1 M NH_4Ac.

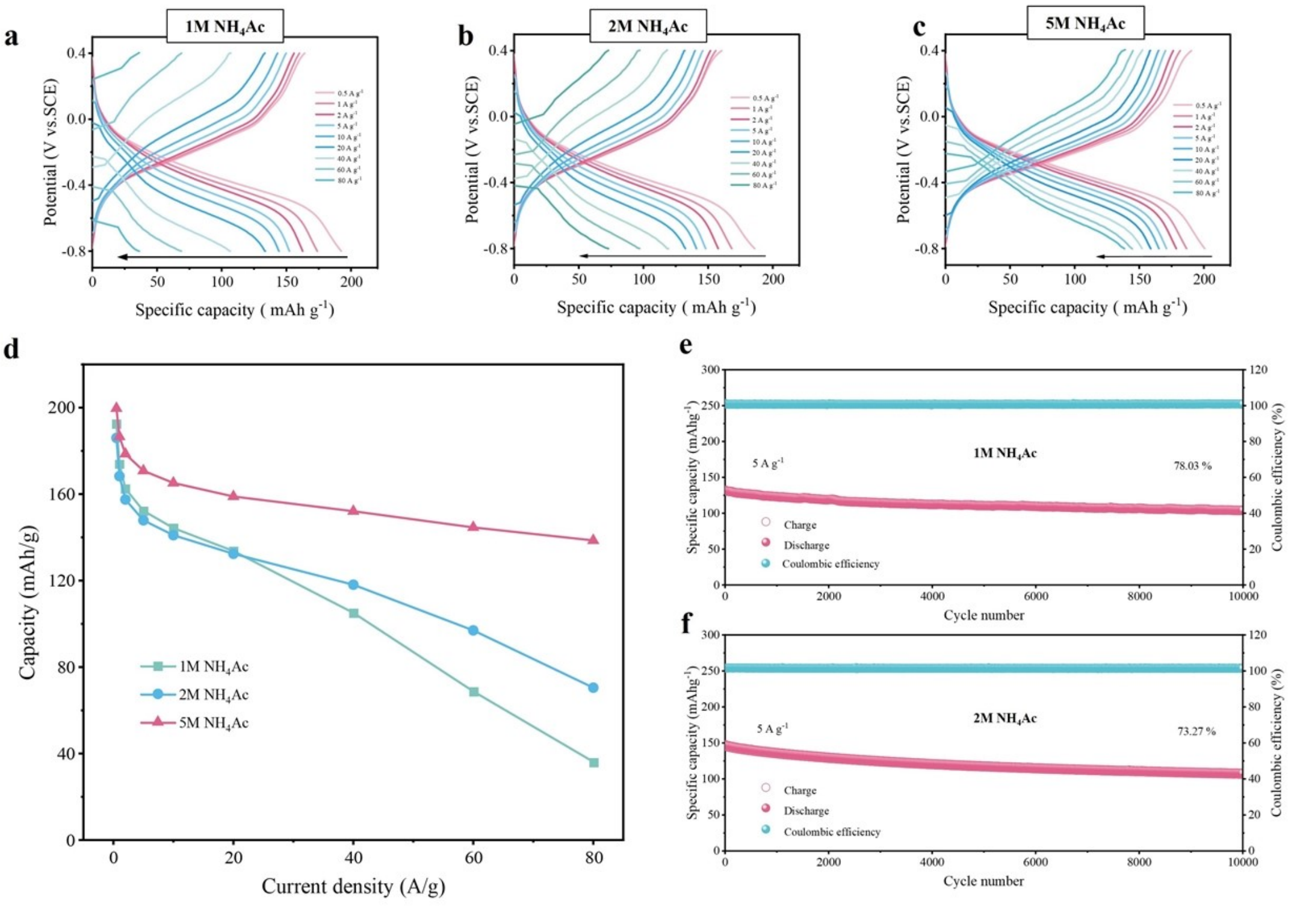

Fig. S8 (a−c) Rate performance of HMND electrode with 1 M NH_4Ac, 2 M NH_4Ac, and 5 M NH_4Ac, (d) Comparison of the rate performance of the HMND electrode in 1 M, 2 M, and 5 M NH_4Ac electrolytes, (e−f) Cycling stability of HMND electrode at 5 A g^{-1} in 1 M NH_4Ac and 2 M NH_4Ac.

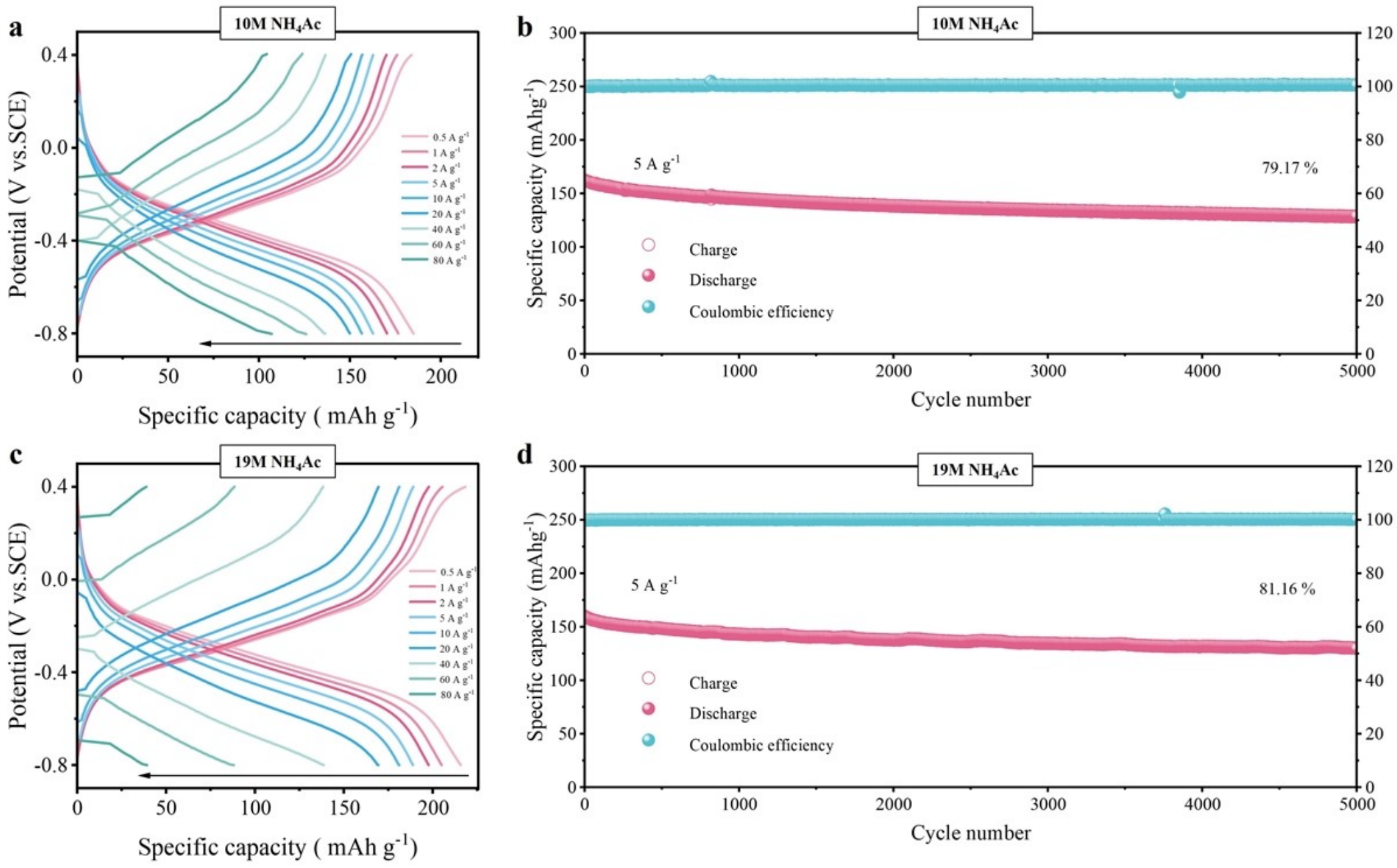

Fig. S9 (a, c) Rate performance of HMND electrode with 10 M NH_4Ac and 19 M NH_4Ac, (b, d) Cycling stability of HMND electrode at 5 A g^{-1} in 10 M NH_4Ac and 19 M NH_4Ac.

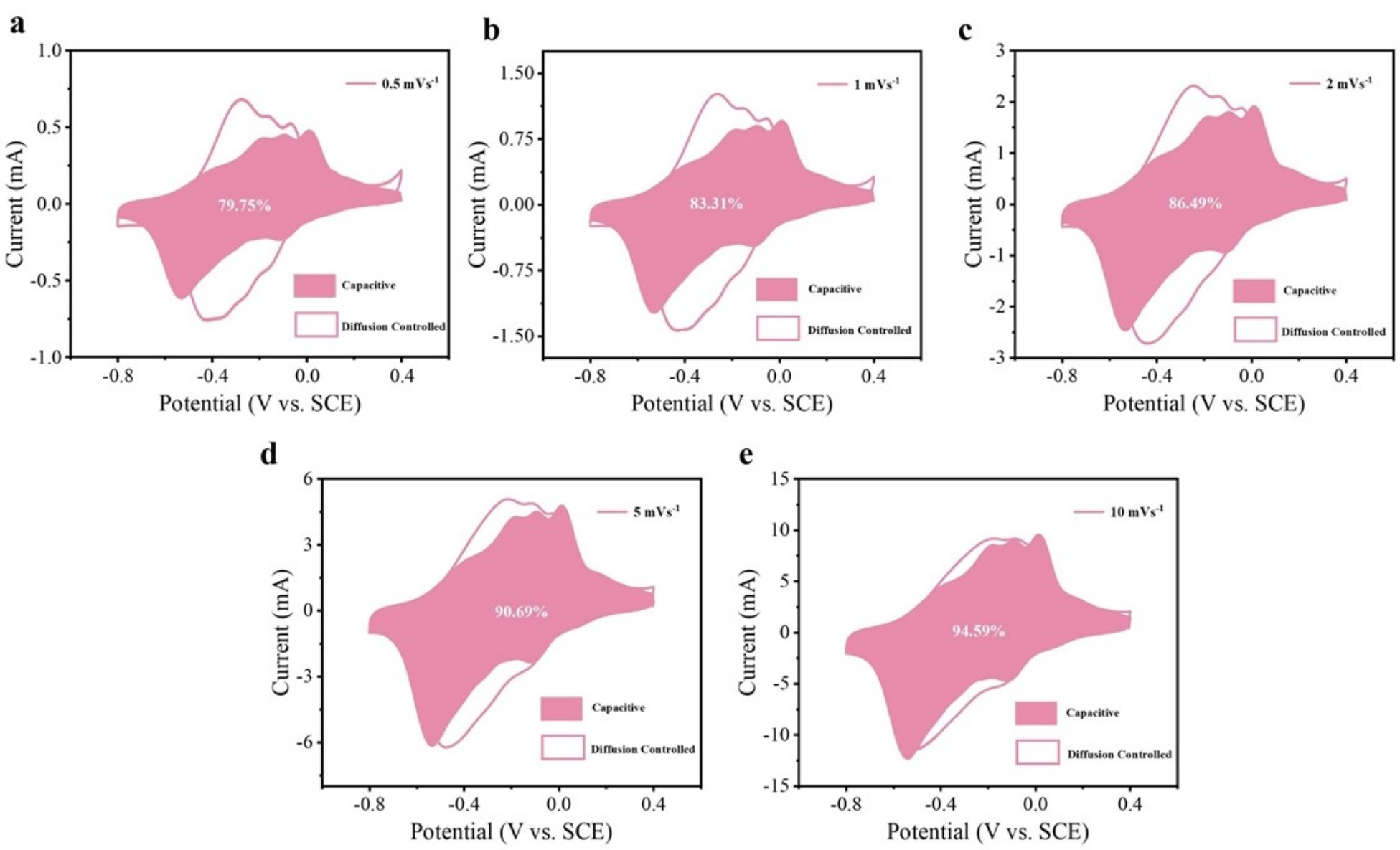

Fig. S10 Capacitance contribution at 0.5 mV s^{-1} (a), 1 mV s^{-1}(b), 2 mV s^{-1}(c), 5 mV s^{-1}(d), 10 mV s^{-1}(e) of HMND in 5 M NH_4Ac solution.

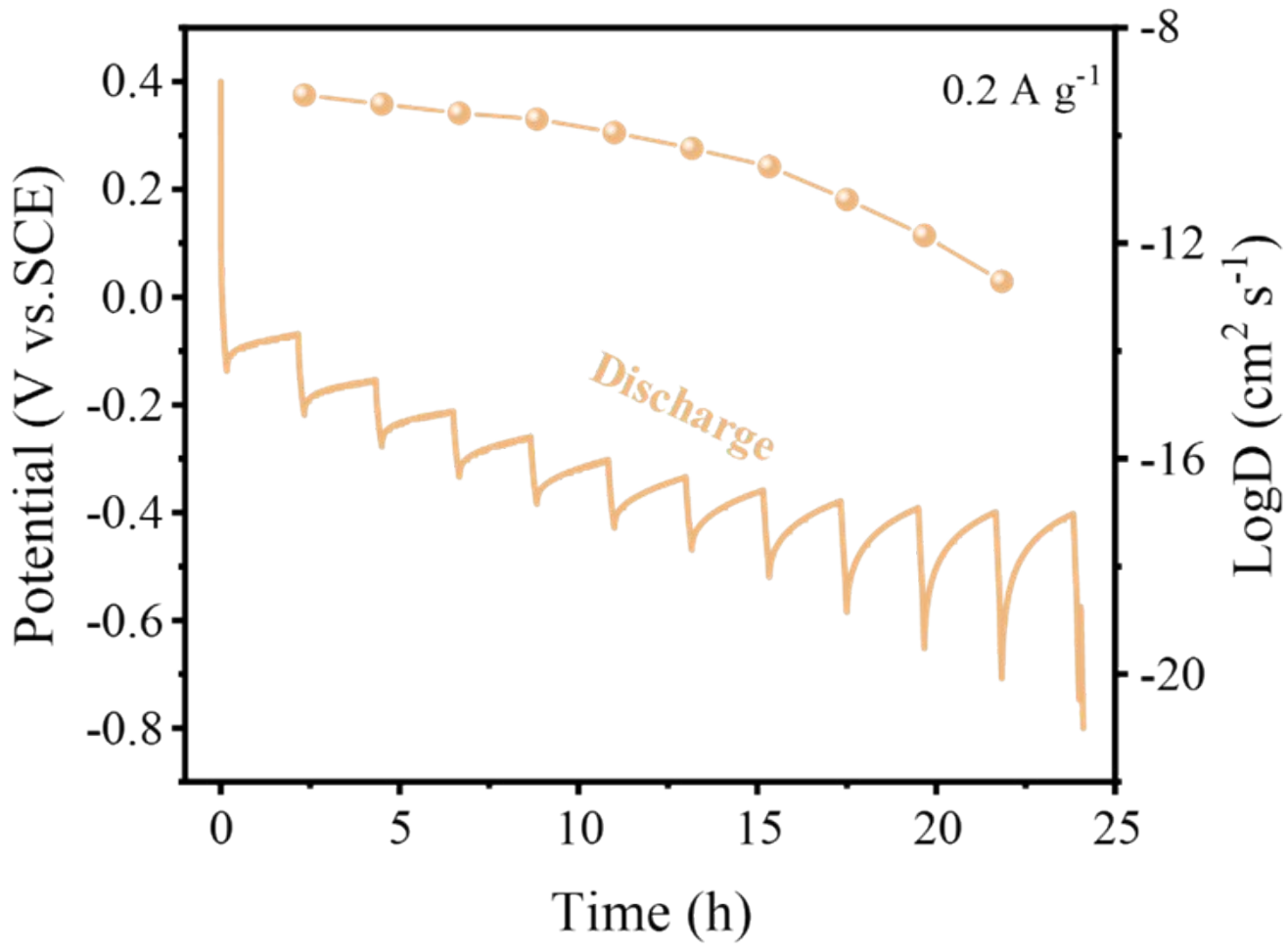

Fig. S11 Discharge GITT Curves with ion diffusion coefficients.

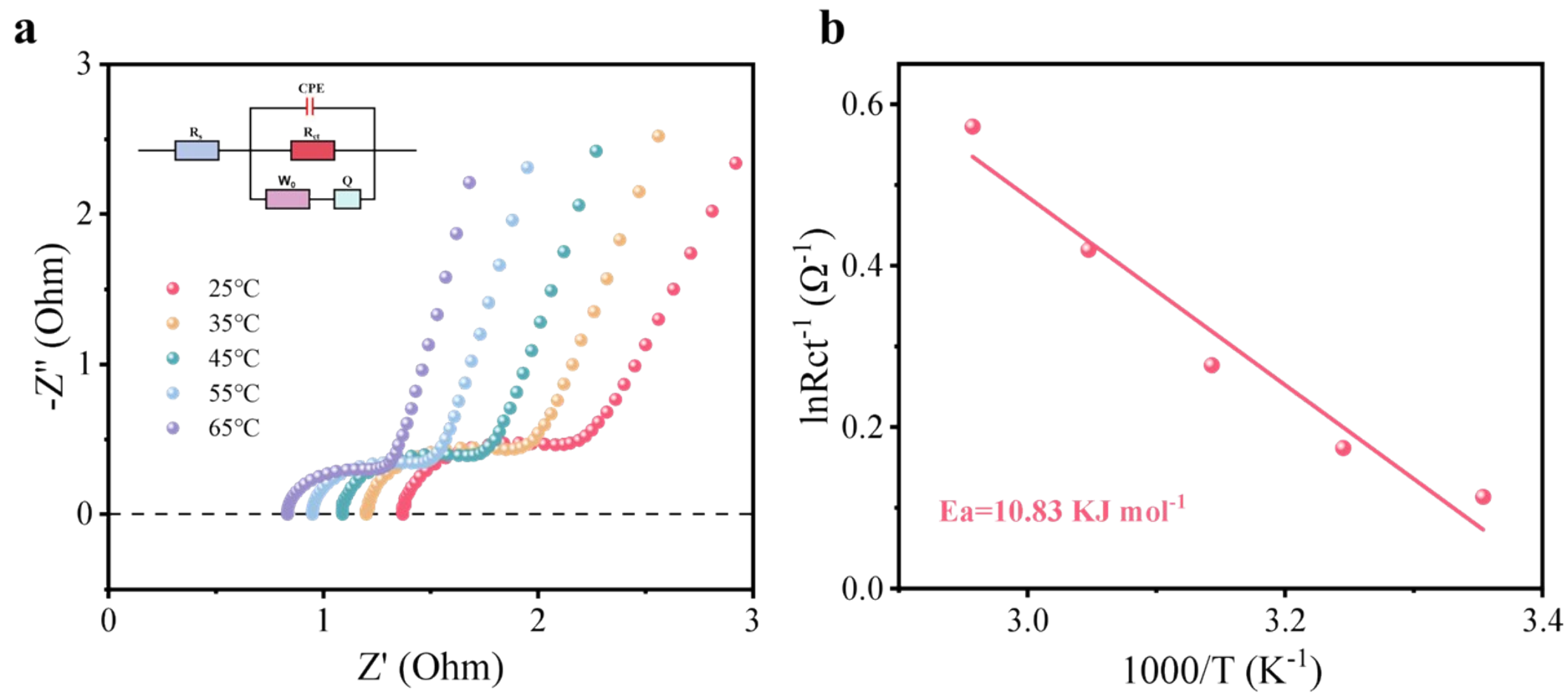

Fig. S12 (a) Nyquist plots of HMND in NH_4Ac electrolyte measured at different temperatures, (b) Calculated E_a values.

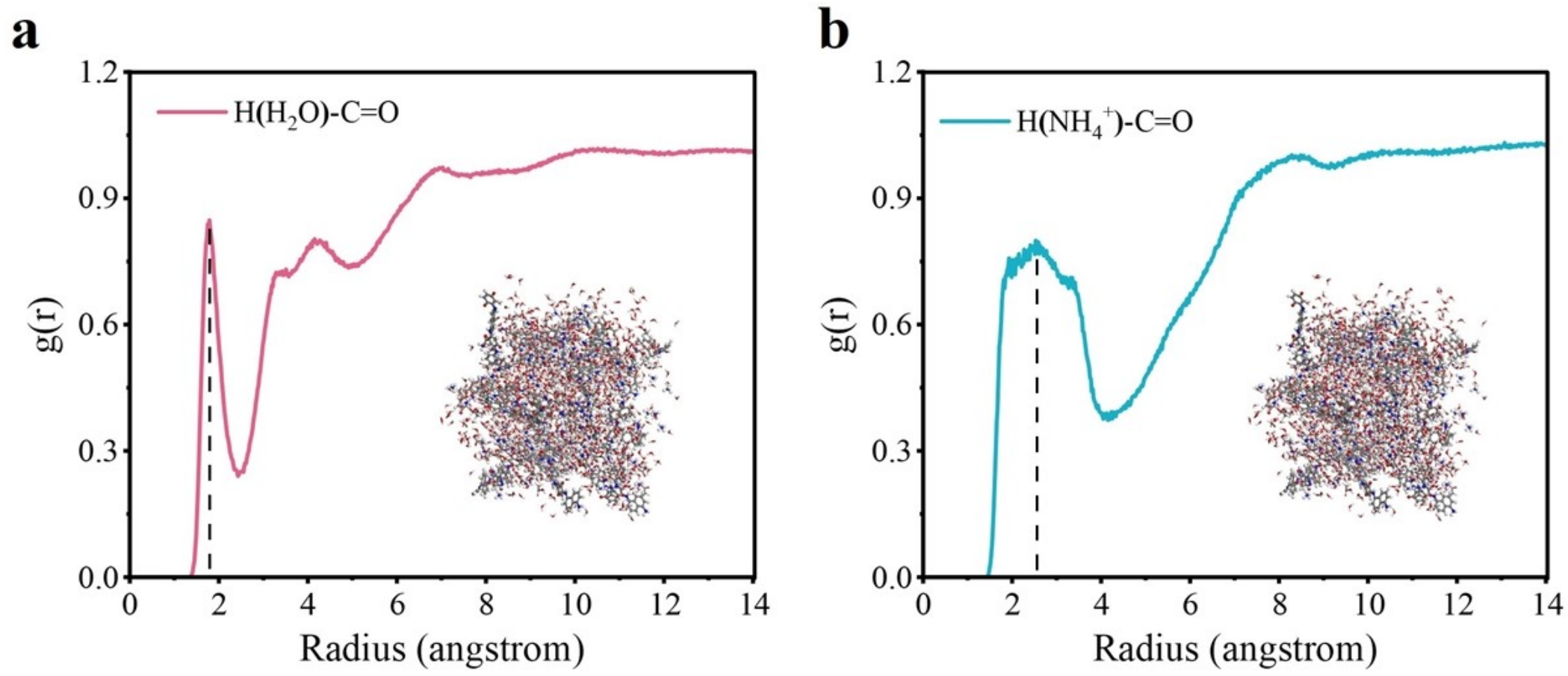

Fig. S13 Radial distribution functions (RDFs) of HMND. (a) RDF between H_2O and the C=O of HMND. (b) RDF between NH_4^+ and the C=O of HMND.

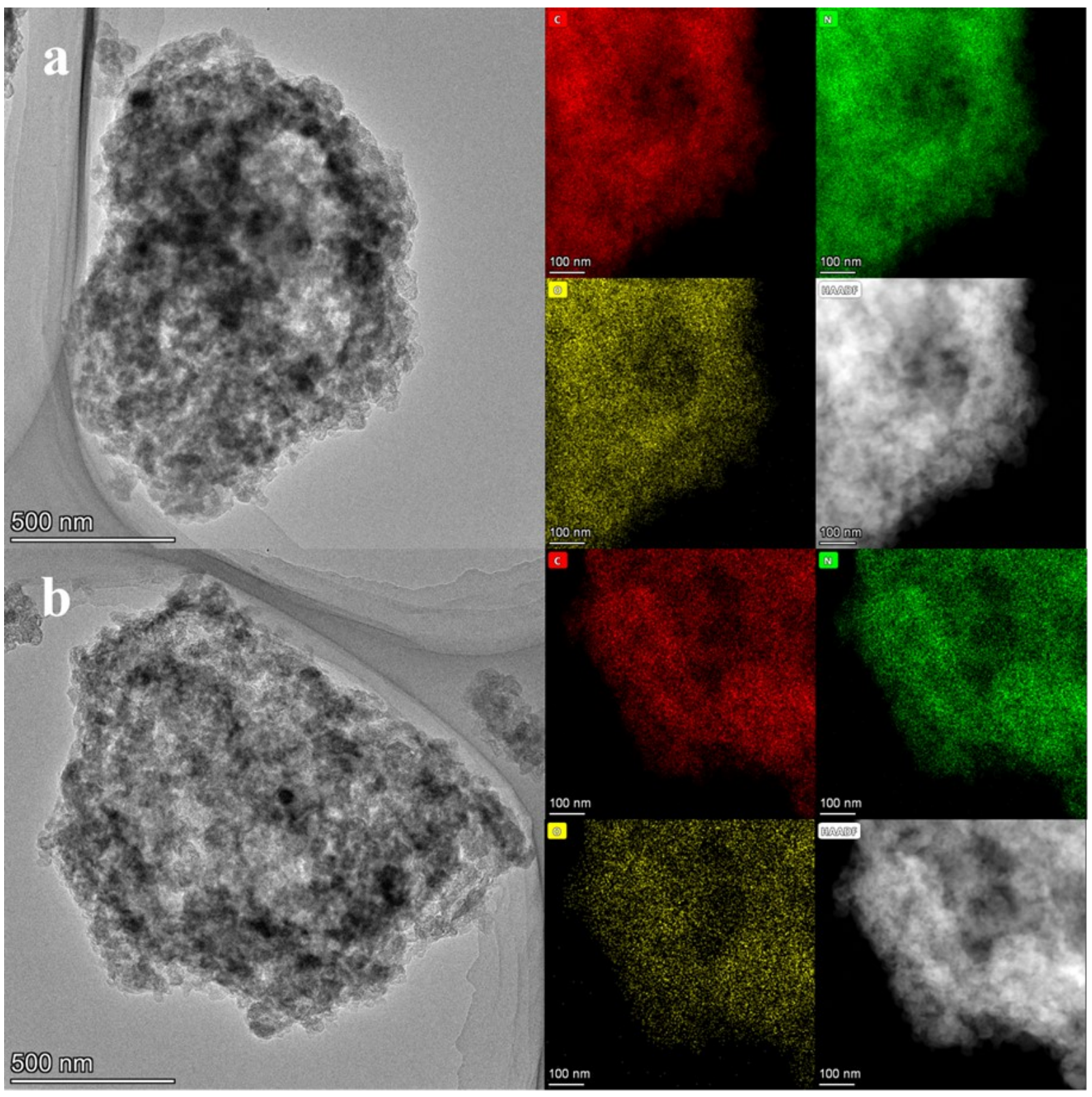

Fig. S14 TEM-EDS mapping images of the HMND electrodes (a) before and (b) after 10000 cycles.

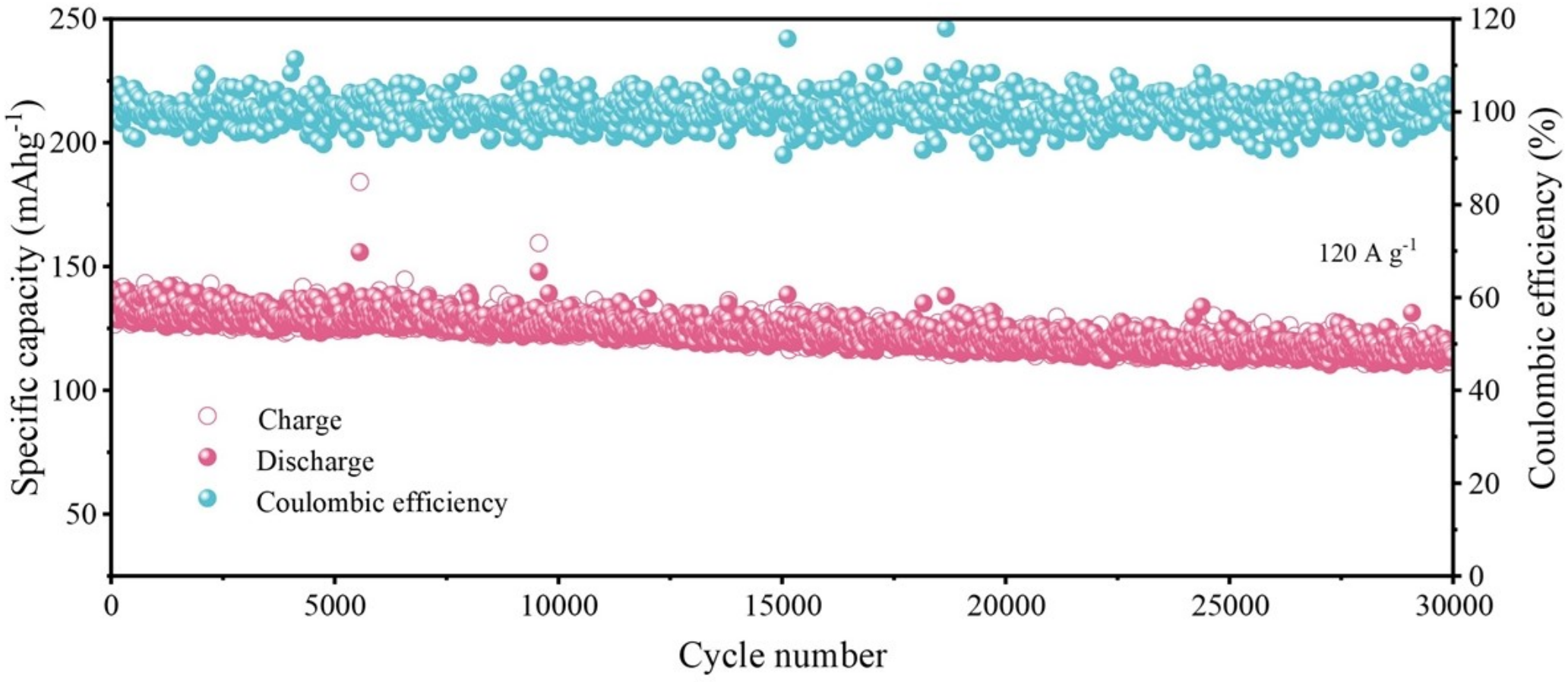

Fig. S15 Cycling performance of HMND at a current density of 120 A g^{-1}.

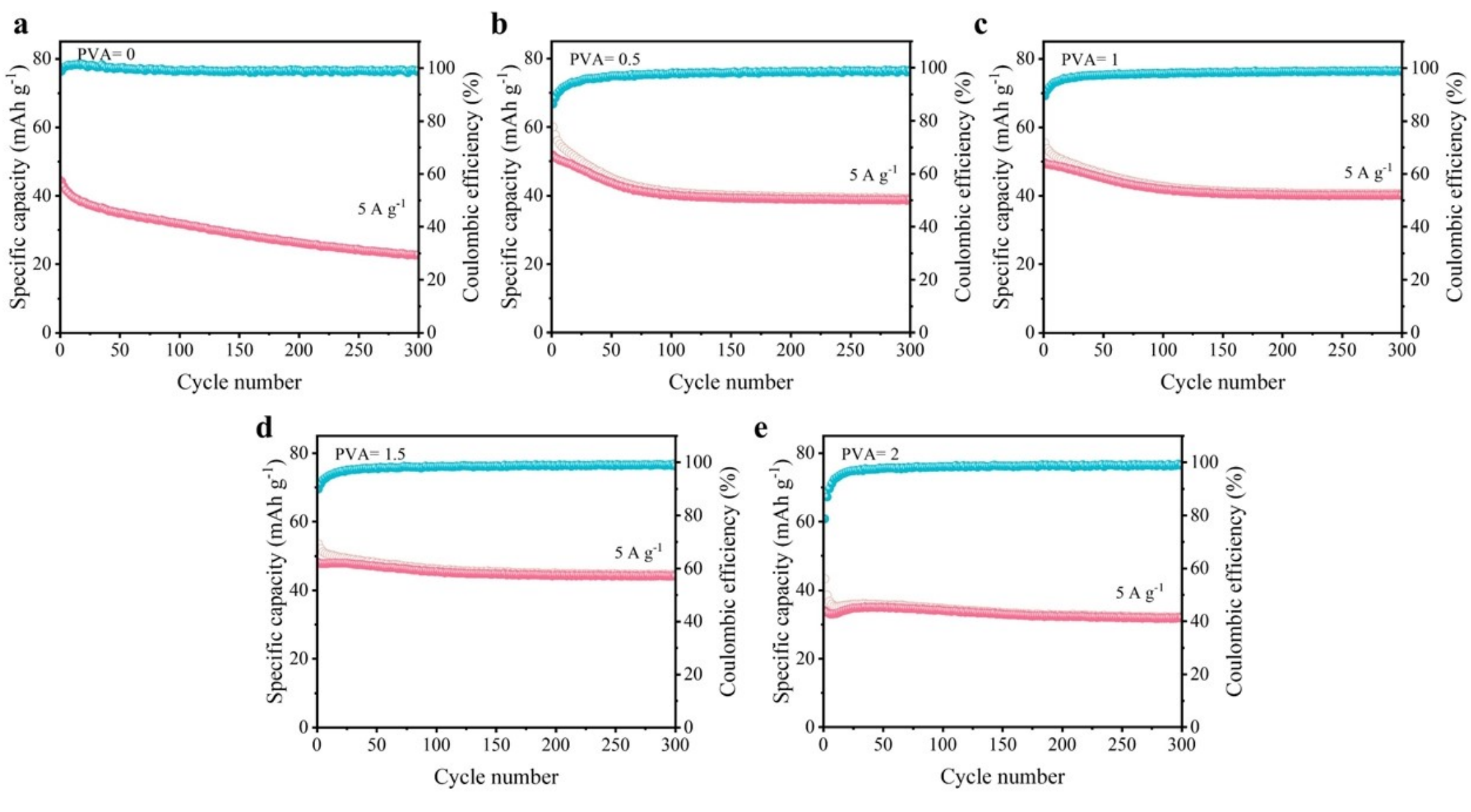

Fig. S16 Cycling performance of MnO@C with (a) PVA=0, (b) PVA=0.5, (c) PVA=1, (d) PVA=1.5, (e) PVA=2 at 5 A g^{-1}.

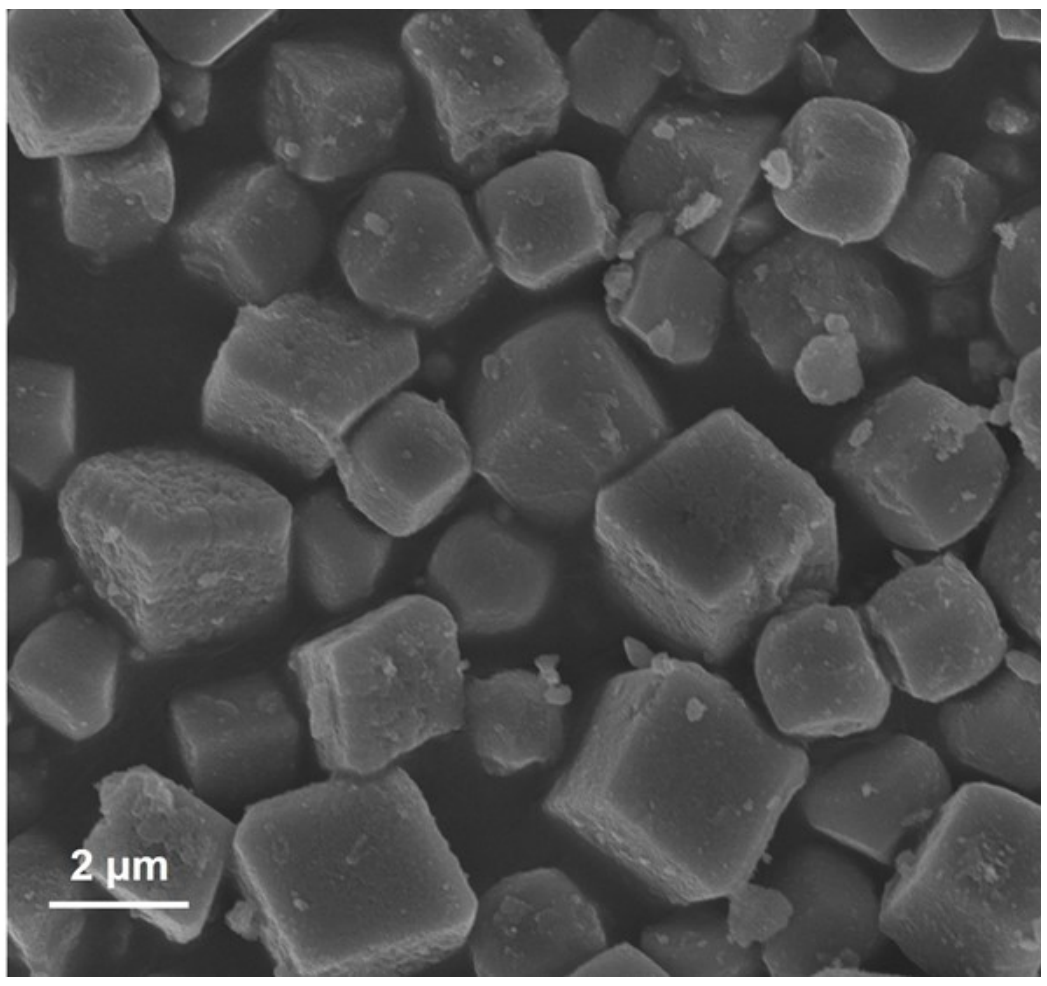

Fig. S17 SEM image of MnO@C.

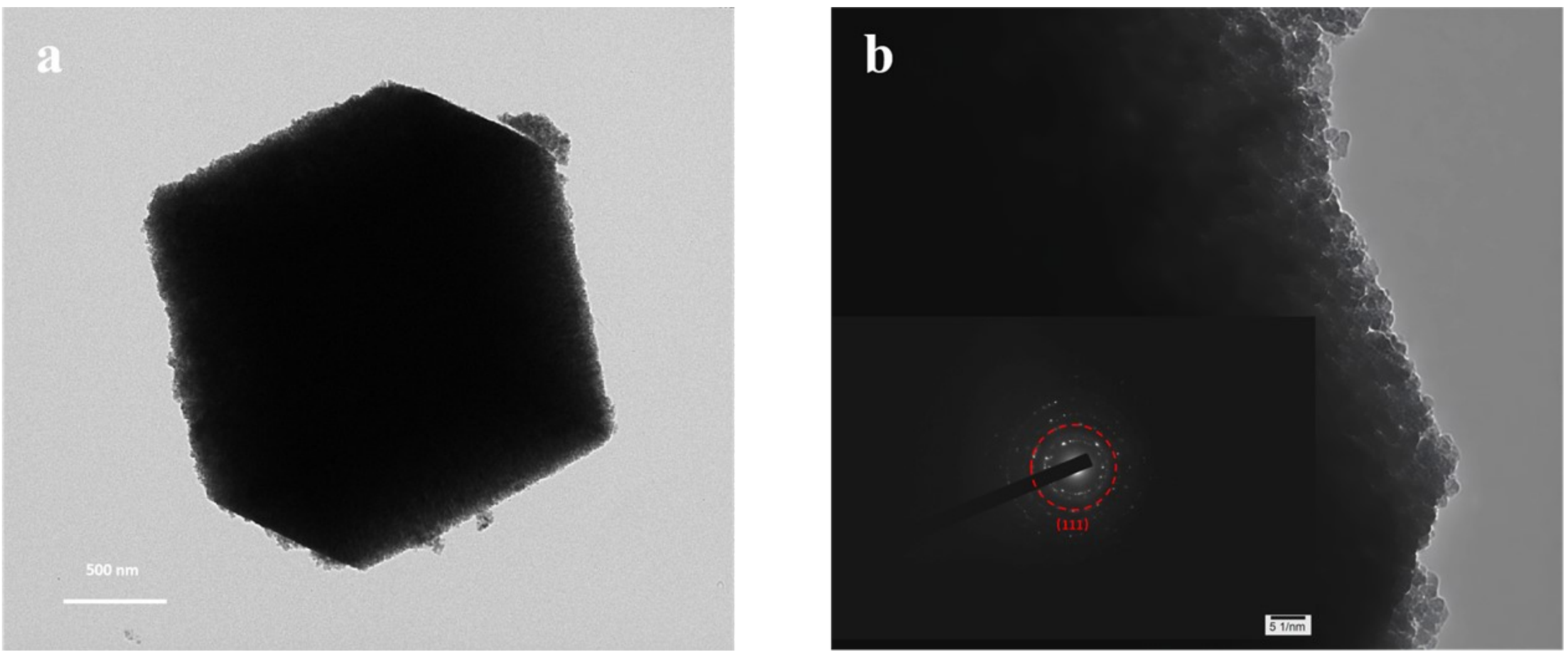

Fig. S18 (a) HAADF-STEM, (b) TEM image and SAED pattern of MnO@C.

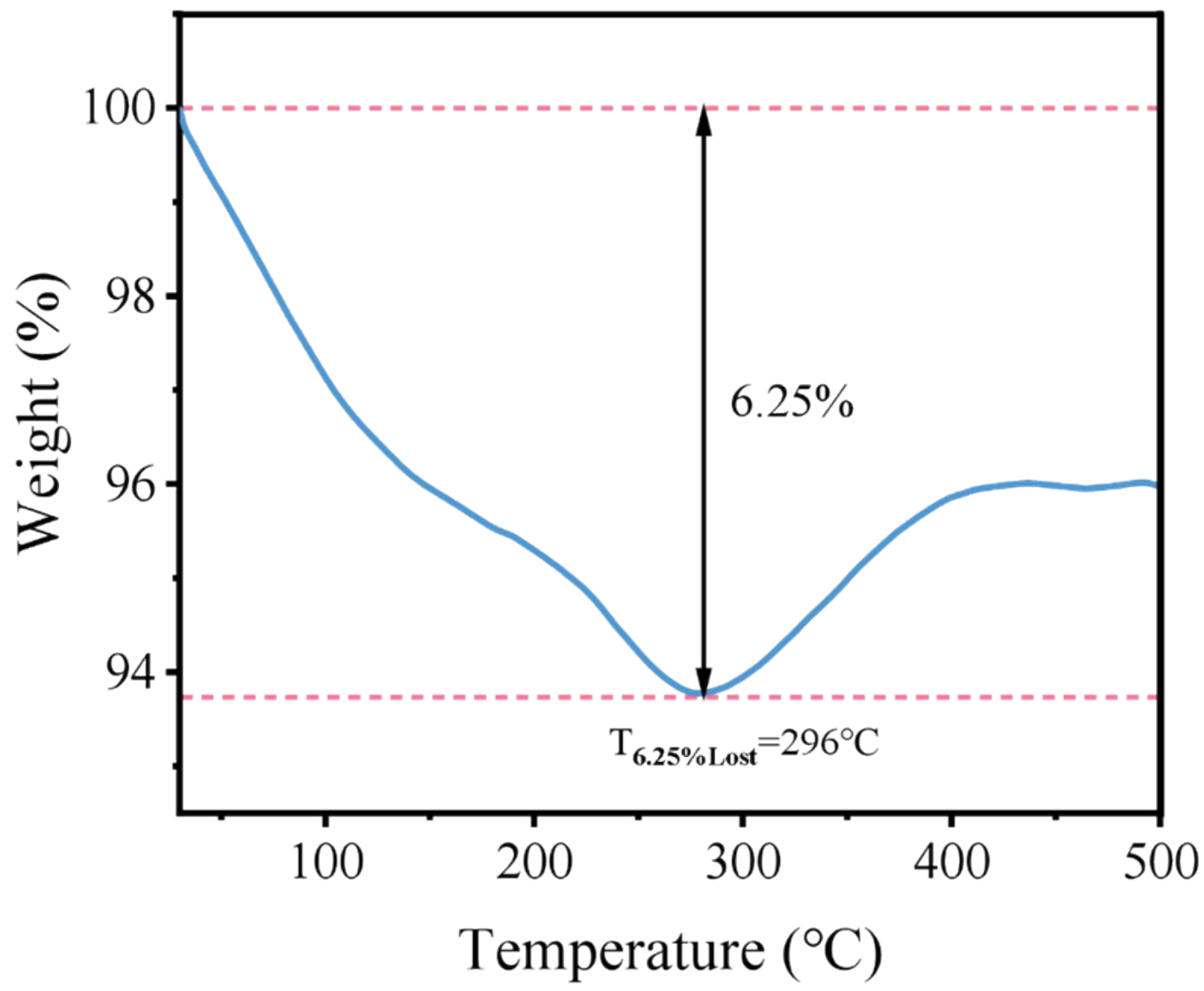

Fig. S19 TGA curve for MnO@C.

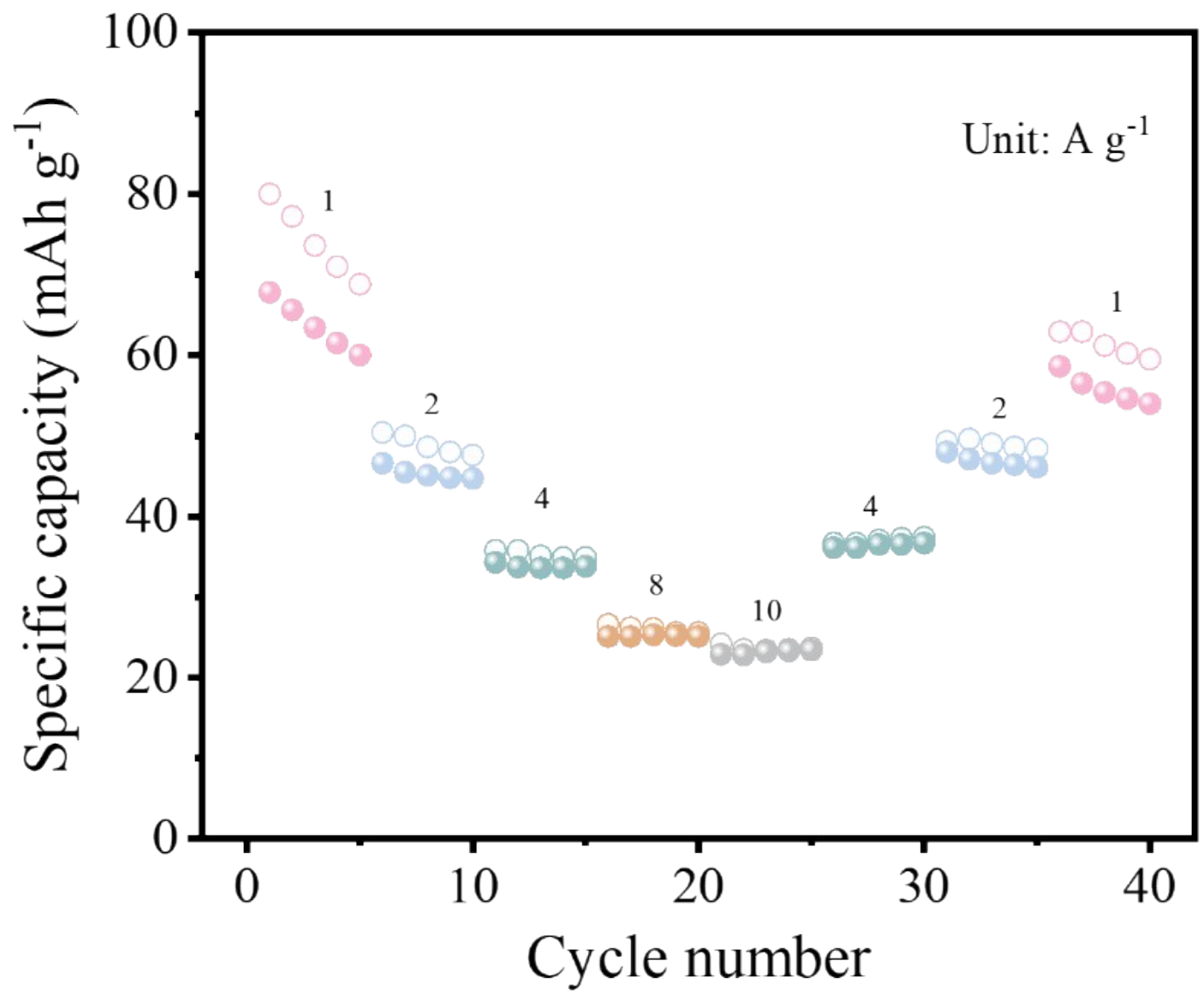

Fig. S20 Rate performance of MnO@C.

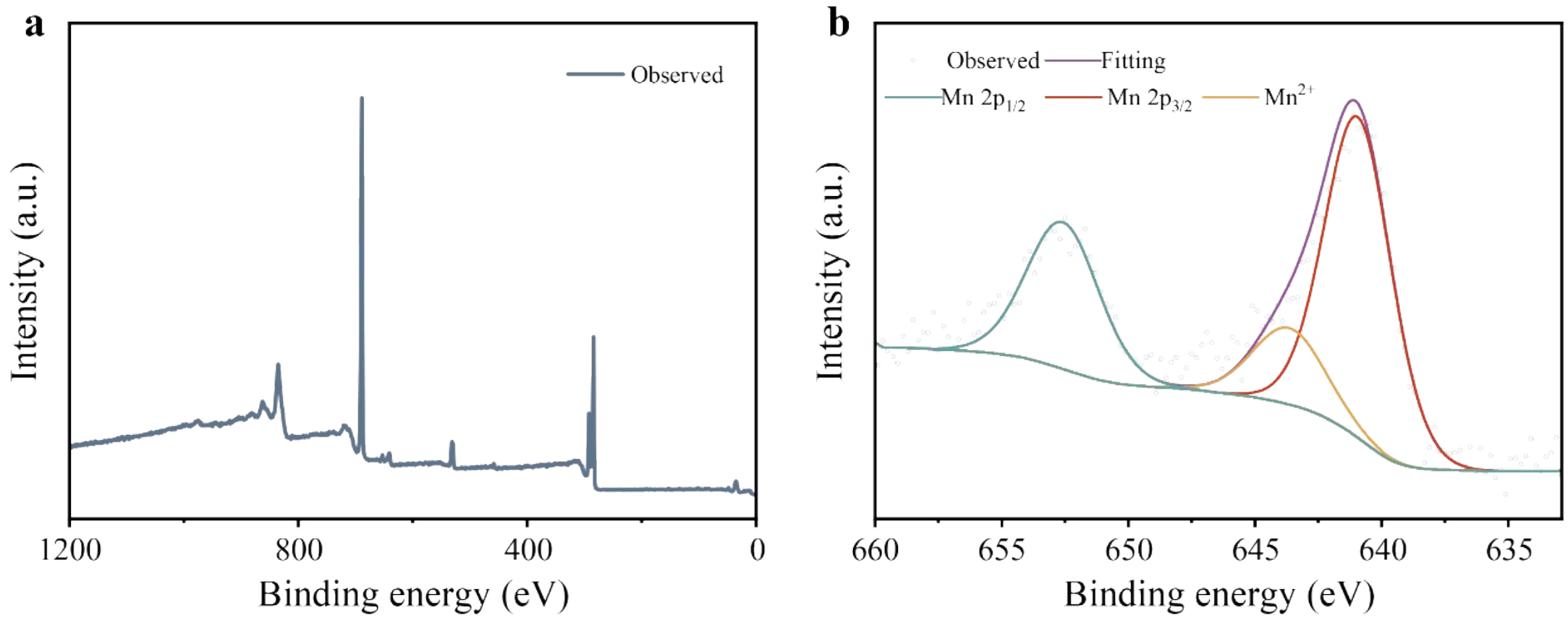

Fig. S21 (a) XPS survey spectrum of MnO@C**,** (b) XPS spectra of Mn 2p (pristine).

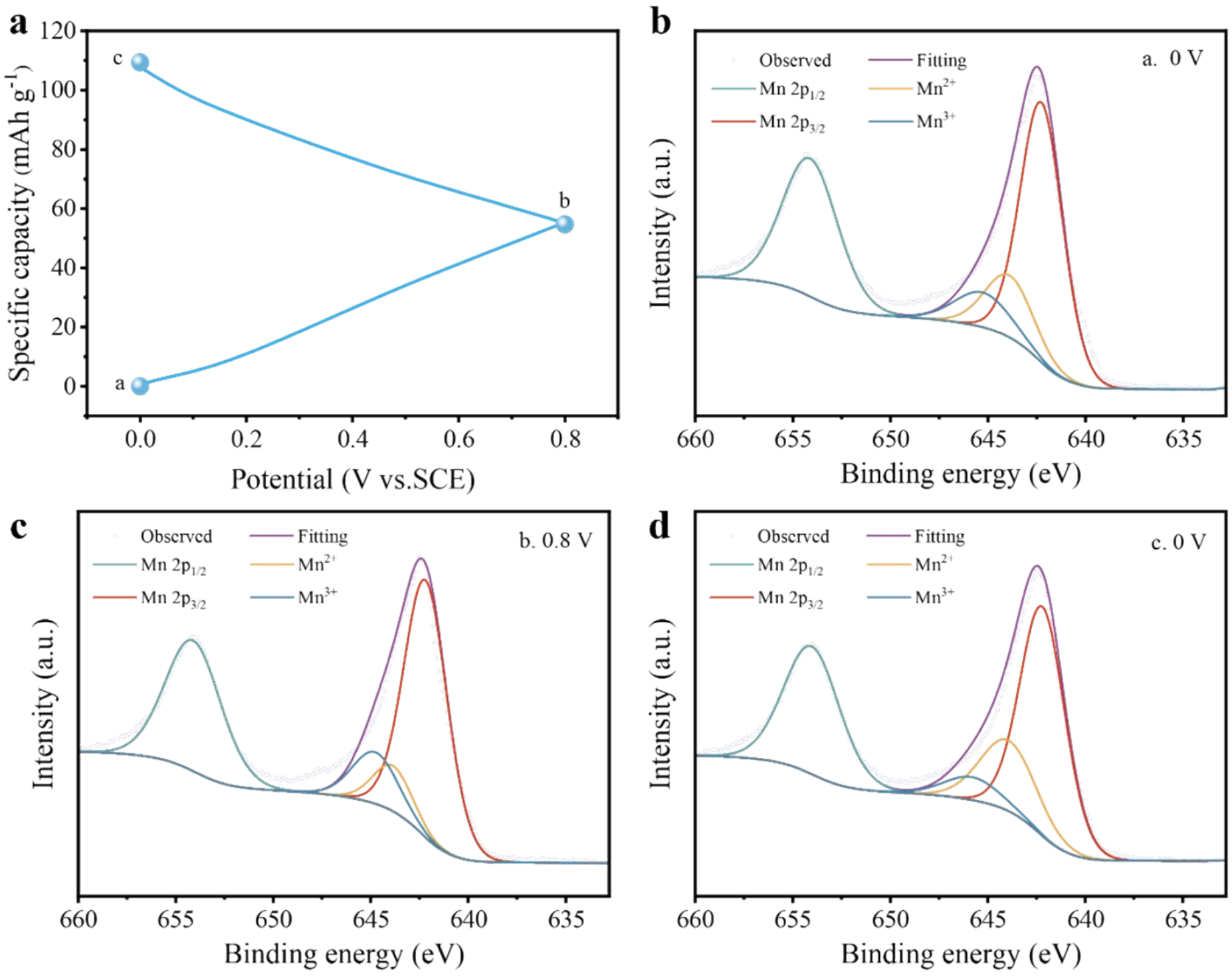

Fig. S22 (a) Ex-situ high-resolution XPS spectra of MnO@C corresponding to the points at different potentials, Mn 2p in pristine (b), fully charged (c) and discharge state (d) of MnO@C electrode.

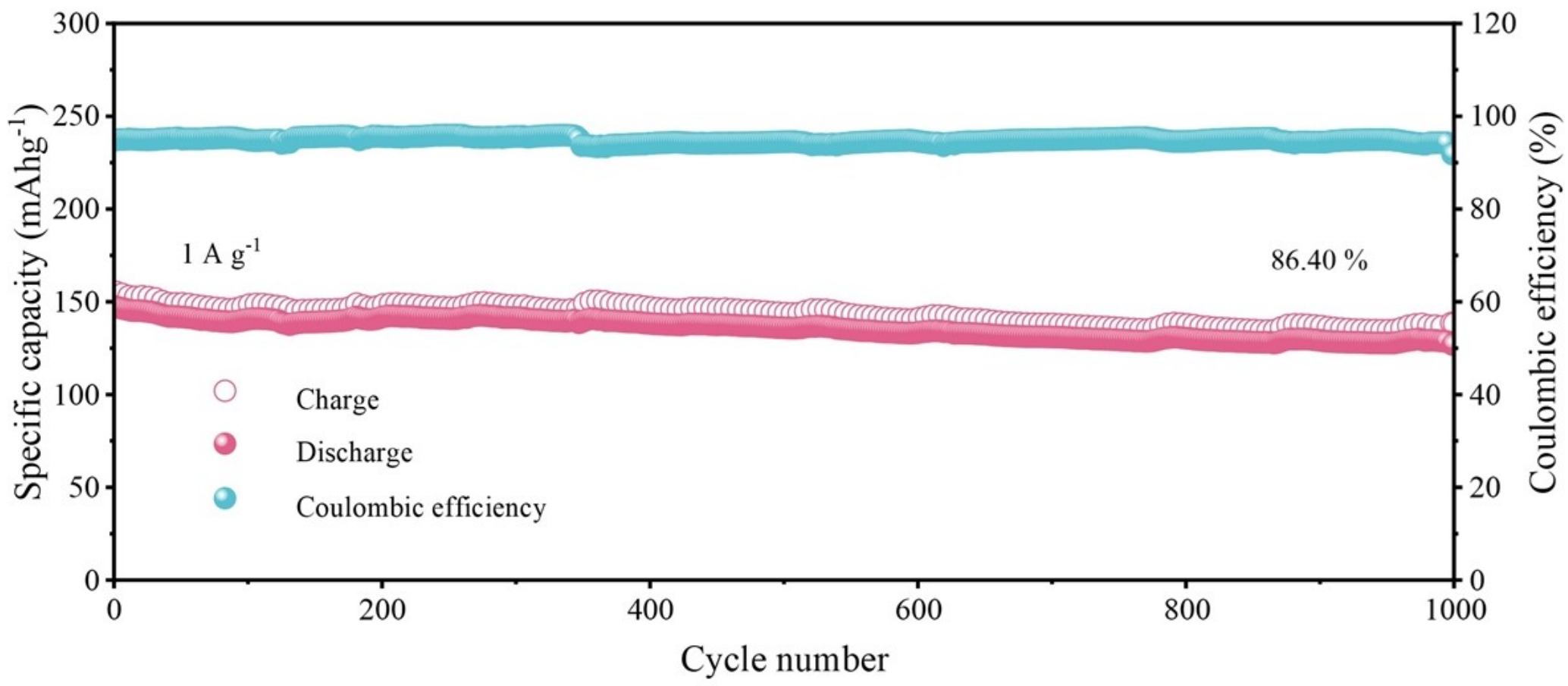

Fig. S23 Cycling performance of the full battery at a current density of 1 A g^{-1}.

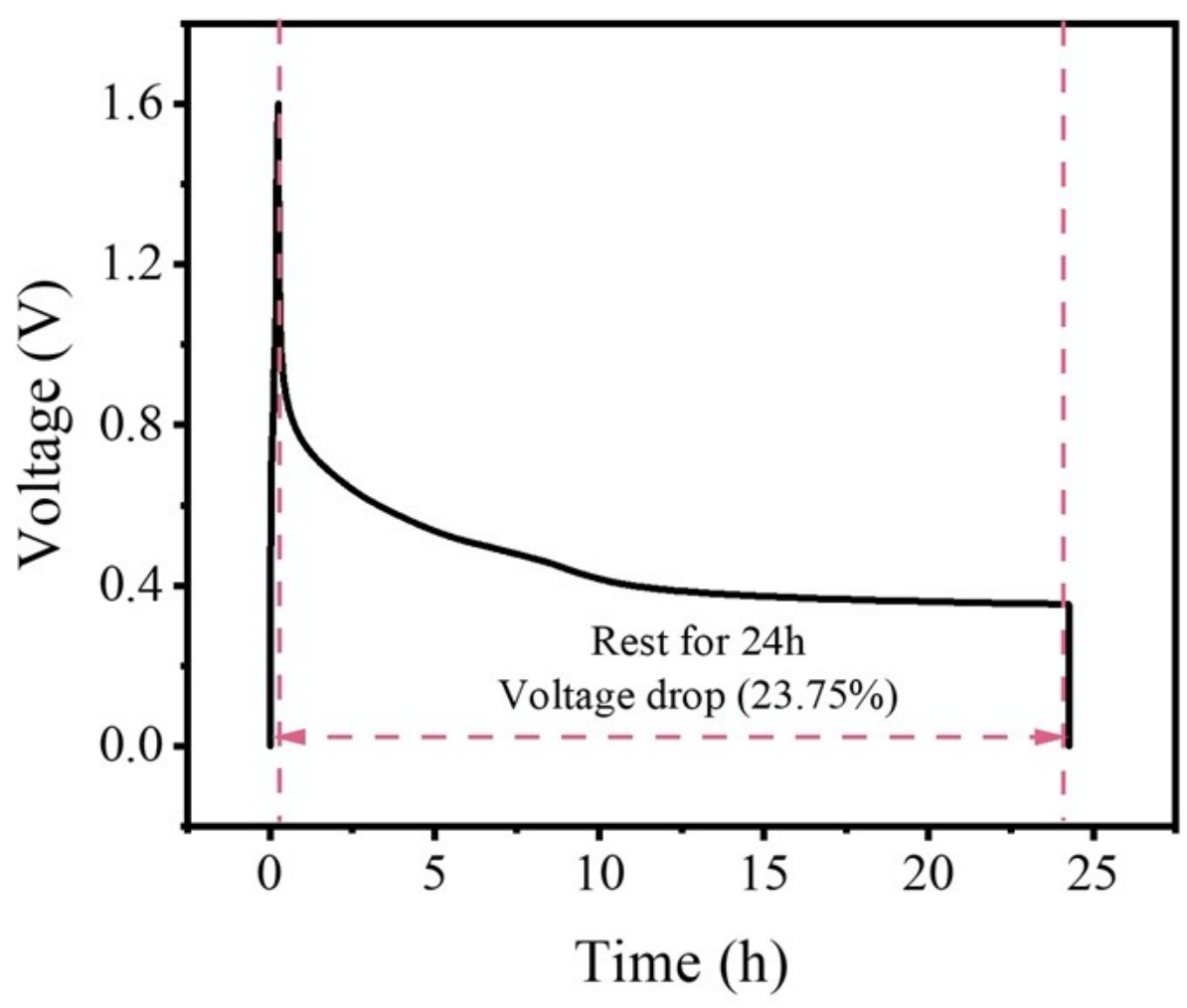

Fig. S24 Self-Discharge of the full cell.

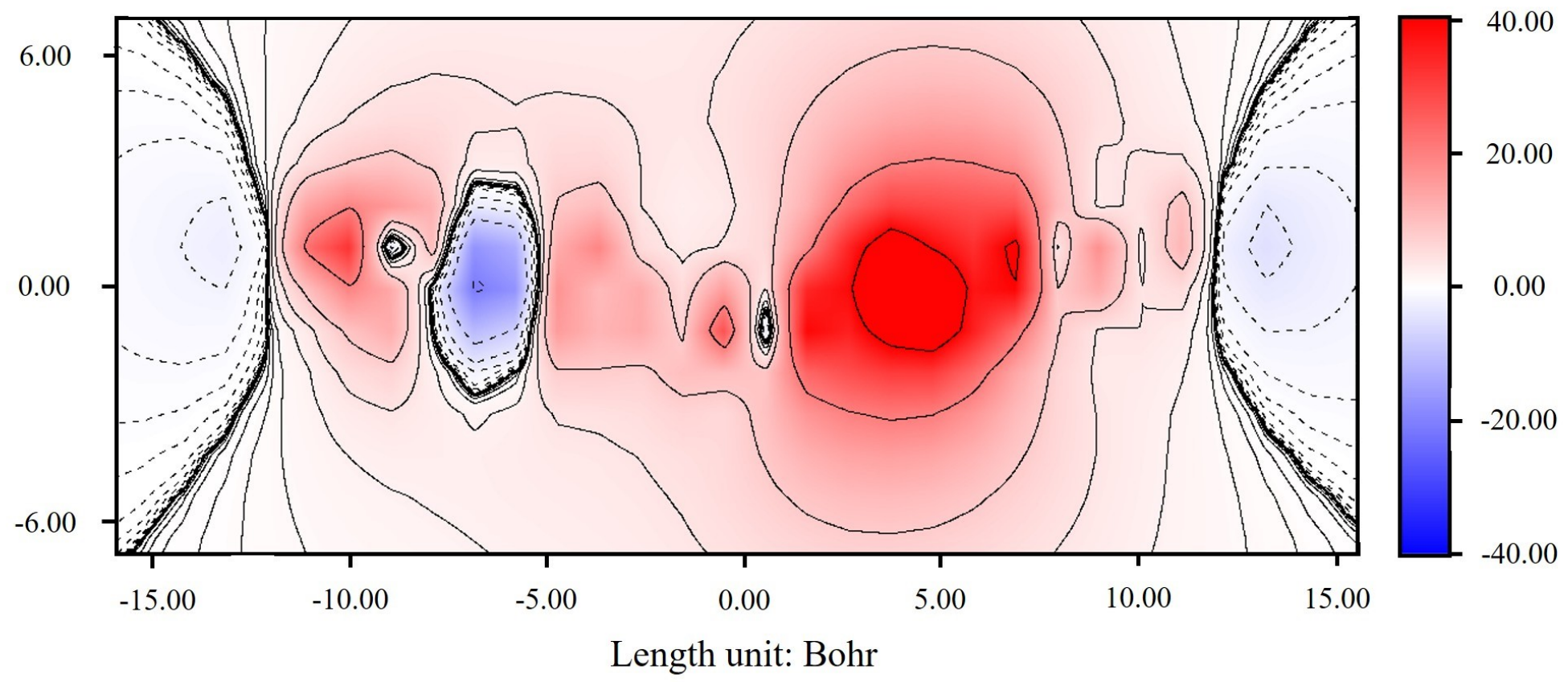

Fig. S25 Color-filled contour map of ICSSZZ on the XZ cross-section perpendicular to the symmetry plane of the HMND molecule.

Table S1. Summary of R_Ω and ionic conductivities for the three ammonium-based electrolytes.

Electrolyte	R_Ω	Conductivity (mS cm^{-1})
1 M NH_4Ac	6.9	160.92
1 M NH_4Cl	5.8	163.49
0.5 M $(NH_4)_2SO_4$	5.4	143.54

Table S2. The comparison of rate capability of HMND anode with other reported advanced materials.

Cathode	The capacity (highest current density)	Times of The same current	Capacity retention minimum / maximum	Ref.
HATP-PT COF	74 mAh g^{-1} at 10 A g^{-1}	50 times of 0.2 A g^{-1}	68.2%	[11]
MXene PANI	76.2 mAh g^{-1} at 5 A g^{-1}	50 times of 0.1 A g^{-1}	31.1%	[12]
DAAQ-TP-COF	51 mAh g^{-1} at 10 A g^{-1}	100 times of 0.1 A g^{-1}	36.2%	[13]
V_2CT_x MXene	53 mAh g^{-1} at 5 A g^{-1}	5 times of 1 A g^{-1}	61.5%	[14]
PNNI	110 mAh g^{-1} at 5 A g^{-1}	50 times of 0.1 A g^{-1}	45.7%	[15]
ploy(1,5-NAPD)	47 mAh g^{-1} at 50 A g^{-1}	50 times of 1 A g^{-1}	33.3%	[16]
HATNTN	81.67 mAh g^{-1} at 20 A g^{-1}	20 times of 1A g^{-1}	40.15%	[17]
HAQ	78 mAh g^{-1} at 5 A g^{-1}	25 times of 0.2 A g^{-1}	38.8%	[18]
PTNQ	57.2 mAh g^{-1} at 20 A g^{-1}	10 times of 2 A g^{-1}	33.5%	[19]
$Nb_2Mo_3O_{14}$	69 mAh g^{-1} at 2 A g^{-1}	10 times of 0.2 A g^{-1}	30.7%	[20]
PTAQ	118 mAh g^{-1} at 15 A g^{-1}	15 times of 1 A g^{-1}	54.9%	[21]
**HMND**	**116.3 mAh g^{-1} at 120 A g^{-1}**	**240 times of 0.5 A g^{-1}**	**57.9%**	**This work**

Table S3. The comparison of rate capability of MnO@C cathode with other reported advanced materials.

Cathode	Electrolyte	The capacity (highest current density)	The max capacity (current density)	Ref.
CuHCF	2 M NH_4OTf 3 M Sucrose	30 mAh g^{-1} at 10 A g^{-1}	56 mAh g^{-1} at 0.2 A g^{-1}	[11]
Ni-APW	1 M NH_4Ac	28 mAh g^{-1} at 2 A g^{-1}	60 mAh g^{-1} at 0.1 A g^{-1}	[15]
NiHCF	19 M NH_4Ac	15 mAh g^{-1} at 10 A g^{-1}	44 mAh g^{-1} at 1 A g^{-1}	[16]
α-MnO_2	1 M NH_4Ac	30 mAh g^{-1} at 2 A g^{-1}	72 mAh g^{-1} at 0.2 A g^{-1}	[18]
MnO@C	**5 M NH_4Ac**	**24 mAh g^{-1} at 10 A g^{-1}**	**58 mAh g^{-1} at 1 A g^{-1}**	**This work**

Table S4. Summary electrochemical performances of HMND// MnO@C-1.5G and previously reported aqueous ammonium-ion batteries.

Materials	Electrolyte	Capacity (max)	Cycling performance (Capacity retention)	Ref.
WO_3||MnO_2	0.5 M $(NH_4)_2SO_4$	106.1 mAh g^{-1} at 1 A g^{-1}	95.4% at 3 A g^{-1} for 5000 cycles	22
HATP-PT||CuHCF	2 M NH_4OTf 3 M Sucrose	97 mAh g^{-1} at 0.4 A g^{-1}	89% at 1 A g^{-1} for 20000 cycles	11
ploy(1,5-NAPD)||NiHCF	19 M NH_4Ac	143 mAh g^{-1} at 1 A g^{-1}	88.55% at 4 A g^{-1} for 500 cycles	16
DAAQ-TP-COF||PANI	1 M NH_4Ac	108 mAh g^{-1} at 0.1 A g^{-1}	81% at 4 A g^{-1} for 1000 cycles	13
MnO_2/CNTs||PANI	1 M $(NH_4)_2SO_4$ 0.1 M H_2SO_4	101.4 mAh g^{-1} at 1 A g^{-1}	75.7% at 1 A g^{-1} for 10000 cycles	23
PTCDI//$Na_{0.7}Al_{0.1}Mn_{0.9}O_2$	2 M $(NH_4)_2SO_4$	104 mAh g^{-1} at 1 A g^{-1}	79% at 1 A g^{-1} for 1000 cycles	24
**HMND//MnO@C-1.5G**	**5 M NH_4Ac**	**151.6 mAh g^{-1} at 1 A g^{-1}**	**81.63% at 5 A g^{-1} for 10000 cycles**	**This work**

Notes and references

1 Z. Lin, H.-Y. Shi, L. Lin, X. Yang, W. Wu, X. Sun, *Nat. Commun,* **2021**, *12*, 4424.

2 Z. Song, L. Miao, L. Ruhlmann, Y. Lv, D. Zhu, L. Li, L. Gan, M. Liu, *Adv. Funct. Mater.,* **2022**, *32*, 2208049.

3 M. Huang, Q. He, J. Wang, X. Liu, F. Xiong, Y. Liu, R. Guo, Y. Zhao, J. Yang, L. Mai, *Angew. Chem. Int. Ed.,* **2023**, *62*, e202218922.

4 T. Wang, S. Li, X. Weng, L. Gao, Y. Yan, N. Zhang, X. Qu, L. Jiao, Y. Liu, *Adv. Energy Mater.,* **2023**, *13*, 2204358.

5 S. Wang, Z. Yuan, X. Zhang, S. Bi, Z. Zhou, J. Tian, Q. Zhang, Z. Niu, Angew. Chem. Int. Ed., 2021, 60, 7056-7060 .

6 T. Lu, F. Chen, *J. Theor. Comput. Chem.,* **2012**, *11*, 163.

7 P. Wyatt, S. Warren, M. McPartlin, T. Woodroffe, *J. Chem. Soc., Perkin Trans., 1,* **2001**, 279.

8 V. Milman, B. Winkler, J. A. White, C. J. Pickard, M. C. Payne, E. V. Akhmatskaya and R. H. Nobes, Int. J. Quantum Chem., 2000, 77, 895–910；

9 J. P. Perdew, A. Ruzsinszky, G. I. Csonka, O. A. Vydrov, G. E. Scuseria, L. A. Constantin and K. Burke, Phys. Rev. Lett., 2008, 100, 136406.

10 J. P. Perdew, K. Burke and M. Ernzerhof, Phys. Rev. Lett., 1996, 77, 3865–3868.

11 Z. Tian, V. S. Kale, S. Thomas, S. Kandambeth, I. Nadinov, Y. Wang, W. Wahyudi, Y. Lei, A. Emwas, M. Bonneau, O. Shekhah, O. M. Bakr, O. F. Mohammed, M. Eddaoudi, H. N. Alshareef, *Adv. Mater.,* **2024**, *36*, 2409354.

12 Y. Li, L. Du, L. Zhang, C. Huang, J. Palisaitis, J. Xu, J. Rosen, J. Jiang, L. Qin, *Adv. Sci.,* **2025**, *12*, e11815.

13 J. Liu, K. Guo, W. Guo, J. Chang, Y. Li, F. Bao, *Angew. Chem.* Int. Ed*.,* **2025**, *137*, e202424494.

14 Z. Bao, C. Lu, Q. Liu, F. Ye, W. Li, Y. Zhou, L. Pan, L. Duan, H. Tang, Y. Wu, L. Hu, Z. Sun, *Nat. Commun,* **2024**, *15*, 1934.

15 S. Zhang, K. Zhu, Y. Gao, D. Cao, *ACS Energy Lett.,* **2023**, *8*, 889.

16 L. Yan, Y. Qi, X. Dong, Y. Wang, Y. Xia, *eScience.,* **2021**, *1*, 212.

17 S. Yang, W. Zhao, Y. Mi, B. Li, Y. Dong, K. Xie, W. Zhao, G. Long, P. Du, *Angew. Chem.* Int. Ed., **2025**, *137*, e202511826.

18 W. Qin, J. Liu, W. Guo, C. Chen, J. Chang, P. Liu, G. Feng, F. Bao, *Adv. Funct. Mater.,* **2025**, e29819.

19 Y. Liu, Y. Zhou, Y. Li, H. Zhou, H. Liu, L. Hu, M. Shi, C. Yan, J. Yang, *Chem. Eng. J.,* **2026**, *532*, 174626.

20 H. Ren, Y. Yang, Z. Cao, L. Shen, H. Shao, X. Zhang, S. Dong, *Adv. Mater.,* **2026**, *38*, e21722.

21 J. Yang, X. Zhao, J. Peng, J. Yang, M. Shi, L. Zhang, C. Yan, *J. Colloid Interface Sci.,* **2025**, *700*, 138339.

22 X. Wen, J. Luo, K. Xiang, W. Zhou, C. Zhang, H. Chen, *Chem. Eng. J.,* **2023**, *458*, 141381.

23 Y. Li, L. Du, L. Zhang, C. Huang, J. Palisaitis, J. Xu, J. Rosen, J. Jiang, L. Qin, *Adv. Sci.,* **2025**, *12*, e11815.

24 C. Cheng, S. Bian, Y. You, Q. Liu, Z. Yang, F. Ye, W. Chen, J. Cheng, X. Chen, Z. Tang, K. Zhu, Y. Wu, L. Hu, *Adv. Mater.,* **2026**, *38*, e12356.
